# A novel human-specific splice isoform alters the critical C-terminus of Survival Motor Neuron protein

**DOI:** 10.1038/srep30778

**Published:** 2016-08-02

**Authors:** Joonbae Seo, Natalia N. Singh, Eric W. Ottesen, Brian M. Lee, Ravindra N. Singh

**Affiliations:** 1 Department of Biomedical Sciences, Iowa State University, Ames, Iowa 50011, USA; 2 Center for Advanced Host Defenses, Immunobiotics & Translational Comparative Medicine (CAHDIT), Iowa State University, Ames, IA 50011, USA

## Abstract

Spinal muscular atrophy (SMA), a leading genetic disease of children and infants, is caused by mutations or deletions of *Survival Motor Neuron 1* (*SMN1*) gene. *SMN2*, a nearly identical copy of *SMN1*, fails to compensate for the loss of *SMN1* due to skipping of exon 7. *SMN2* predominantly produces SMNΔ7, an unstable protein. Here we report exon 6B, a novel exon, generated by exonization of an intronic Alu-like sequence of *SMN*. We validate the expression of exon 6B-containing transcripts *SMN6B* and *SMN6BΔ7* in human tissues and cell lines. We confirm generation of *SMN6B* transcripts from both *SMN1* and *SMN2*. We detect expression of SMN6B protein using antibodies raised against a unique polypeptide encoded by exon 6B. We analyze RNA-Seq data to show that hnRNP C is a potential regulator of *SMN6B* expression and demonstrate that *SMN6B* is a substrate of nonsense-mediated decay. We show interaction of SMN6B with Gemin2, a critical SMN-interacting protein. We demonstrate that SMN6B is more stable than SMNΔ7 and localizes to both the nucleus and the cytoplasm. Our finding expands the diversity of transcripts generated from human *SMN* genes and reveals a novel protein isoform predicted to be stably expressed during conditions of stress.

More than 95% of human genes with two or more exons are alternatively spliced^1^. One of the potential sources of alternative exons are transposable elements, particularly Alu-like sequences that account for ~10% of the human genome^2^,^3^. As per one estimate, ~5% of alternative exons in humans are derived from Alu-like sequences^4^. Alu elements are primate-specific and some Alu-derived exons are expressed only in humans^5^. Alu-derived exons appear to have played an important role in the evolution of primates in general and humans in particular^6^,^7^. More than a third of alternative splicing events in humans generate premature termination codons (PTCs)^8^. In mammalian cells, transcripts carrying PTCs are effectively degraded by nonsense-mediated decay (NMD)^9^. Physiological conditions that alter the expression of NMD-associated factors are known to affect levels of PTC-bearing transcripts, including those harboring Alu-derived exons^10^.

Humans have two nearly identical copies of the *Survival Motor Neuron* gene: *SMN1* and *SMN2* 11. Both *SMN* genes contain 9 exons and code for an identical protein, SMN (Fig. 1A). The major mRNA generated from *SMN1* retains all nine exons and produces full-length (FL) SMN protein. However, *SMN2* predominantly generates an exon 7-skipped (Δ7) transcript due to a deleterious C6U mutation in exon 7, producing a truncated SMNΔ7 protein^12^. Therefore, loss of *SMN1* results in spinal muscular atrophy (SMA), the most common inherited cause of death in infancy^13^,^14^. SMN has been implicated in many processes including snRNP biogenesis, transcription, translation, DNA recombination, signal recognition particle biogenesis, stress granule formation, signal transduction, vesicular transport, and motor neuron trafficking^15^,^16^,^17^,^18^,^19^,^20^,^21^,^22^,^23^,^24^. Consistently, SMN contains several functional domains (Fig. 1A), and mutations within each domain have been associated with SMA^25^. Gemin2 binding and YG domains of SMN are the most conserved regions from yeast to humans (Supplementary Fig. 1)^26^. The alternatively spliced human *SMN* exon 7 is the last coding exon; it contributes a G residue towards the YG domain and defines the critical C-terminus that enables self-association, governs stability and facilitates subcellular localization of SMN^27^,^28^,^29^. Recent reports employing a multi-exon-skipping-detection assay (MESDA) describe the relative abundance of several *SMN* isoforms^30^,^31^. However, none of the currently known isoforms of *SMN* carries an exon derived from an Alu element.

Here we describe a novel exon, exon 6B, generated by exonization of an Alu element within *SMN* intron 6. We validate the expression and stability of the exon 6B-containting transcripts in various human tissues and cells. We examine the expression, stability, Gemin2-interaction and subcellular localization of SMN6B protein. Our findings uncover an important evolutionary event in humans with significance to potential new functions of *SMN* genes.

## Results

### Exonization of an intronic sequence produces a novel *SMN* transcript

**Figure 1:**
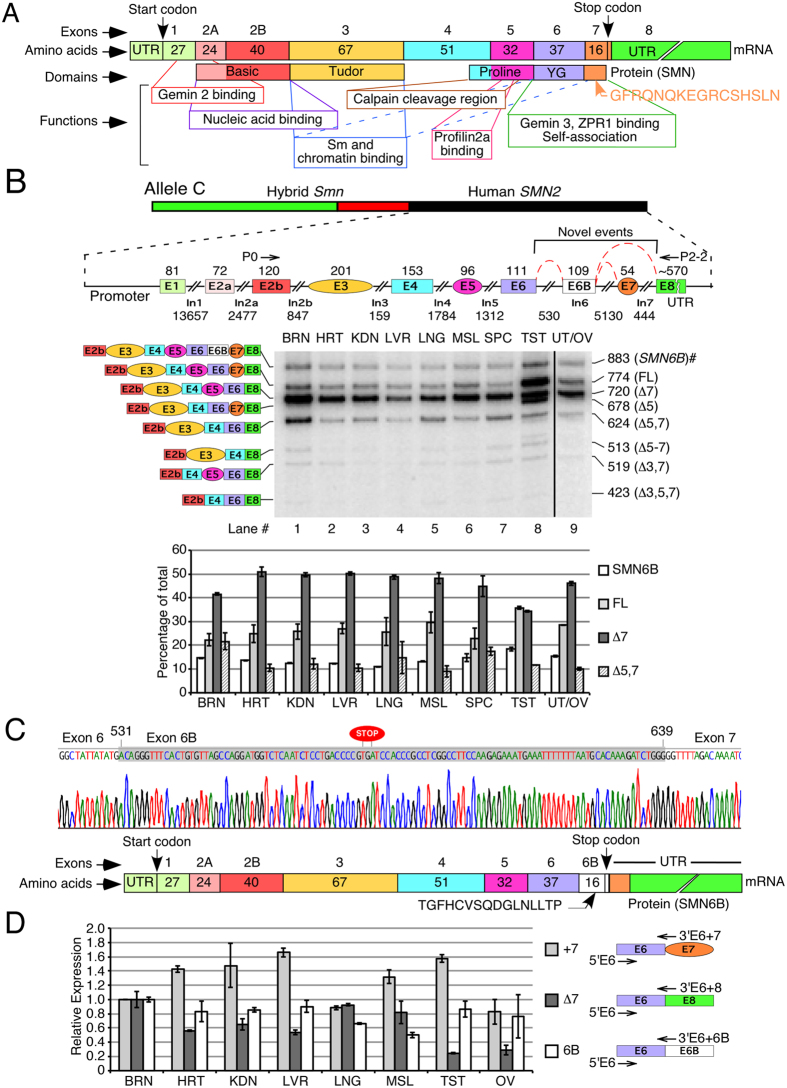
Splicing of human *SMN2* showing inclusion of a novel exon 6B. (**A**) Diagrammatic representation of transcript and protein derived from *SMN* (adapted from Singh *et al.*)^31^. **(B)** Detection of splice isoforms of *SMN2* in various tissues of allele C mice. Top panel shows a diagrammatic representation of allele C transgene. Sizes of exons and introns are given. Annealing positions of primers used for MESDA are shown. Splice variants are indicated on the left of the gel; sizes are indicated on the right. #: novel splice variant [GenBank: KJ780720]. Abbreviations used: BRN, brain; HRT, heart; KDN, kidney; LVR, liver; LNG, lung; MSL, muscle; SPC, spinal cord; TST, testis; UT/OV, uterus/ovaries. Relative abundance of four major splice isoforms (SMN6B, FL, ∆7 and ∆5, 7) is given in the lower panel. **(C)** Portion of cloned DNA sequence confirming insertion of exon 6B (highlighted in gray color) between exons 6 and 7. Numbering starts from the beginning of intron 6. Stop codon in exon 6B is marked. Bottom panel: diagrammatic representation of SMN6B protein. Corresponding exons are indicated at the top. Locations of the start and stop codons, as well as the untranslated regions (UTRs) are marked. **(D)** Relative expression levels of *SMN* splice isoforms in human tissues as determined by QPCR using commercially available RNA. Isoforms and annealing positions of primers are shown to the right. Expression is normalized to total SMN. Error bars represent standard error of three technical replicates.

We employed MESDA to determine the relative abundance of various *SMN2* isoforms in allele C mice, a mild SMA model. Allele C mice harbor a full human *SMN2* gene along with a hybrid *Smn/SMN2* gene at the same locus (Fig. 1B)^32^. We observed Δ7 splice variant as the predominant *SMN2*-derived transcript in all tissues except in testis (Fig. 1B). Higher expression of FL transcripts in testis is due to a recently reported testis-specific splicing switch^30^. Surprisingly, we also observed expression of a large transcript (see 883-nt band in Fig. 1B; GenBank: KJ780720) in all tissues including neuronal, non-neuronal and reproductive tissues/organs. Sequencing of this novel transcript showed that it contained a 109-nt long “exonized” portion of intron 6 (Fig. 1C). We term this sequence as exon 6B, since it is located between exons 6 and 7. For the sake of clarity, we call the novel transcript harboring all *SMN* exons including exon 6B as *SMN6B*. Expression of *SMN6B* varied in different tissues, suggesting a tissue-specific regulation of this transcript (Fig. 1B). In particular, level of *SMN6B* was comparable to FL transcript in brain and spinal cord.

We next investigated the expression of exon 6B-containing transcripts in human tissues from healthy individuals. To capture all exon 6B-containing transcripts of *SMN1* and *SMN2*, we performed real-time QPCR employing forward and reverse primers that annealed to exon 6 and exon 6/exon 6B junction, respectively. In parallel, we examined exon 7-included as well as exon 7-skipped transcripts in these tissues. We normalized the expression levels of transcripts in various tissues to total *SMN* transcript to capture the proportion of mRNA corresponding to each isoform, using expression in brain as standard. Similar to allele C mice, we detected expression of exon 6B-containing transcripts in all human tissues/organs examined. The relative expression of 6B-containing transcripts was similar in most tissues studied, with highest expression in brain and lowest in skeletal muscle (Fig. 1D).

### Exon 6B-containing transcripts are expressed from both *SMN* genes

**Figure 2:**
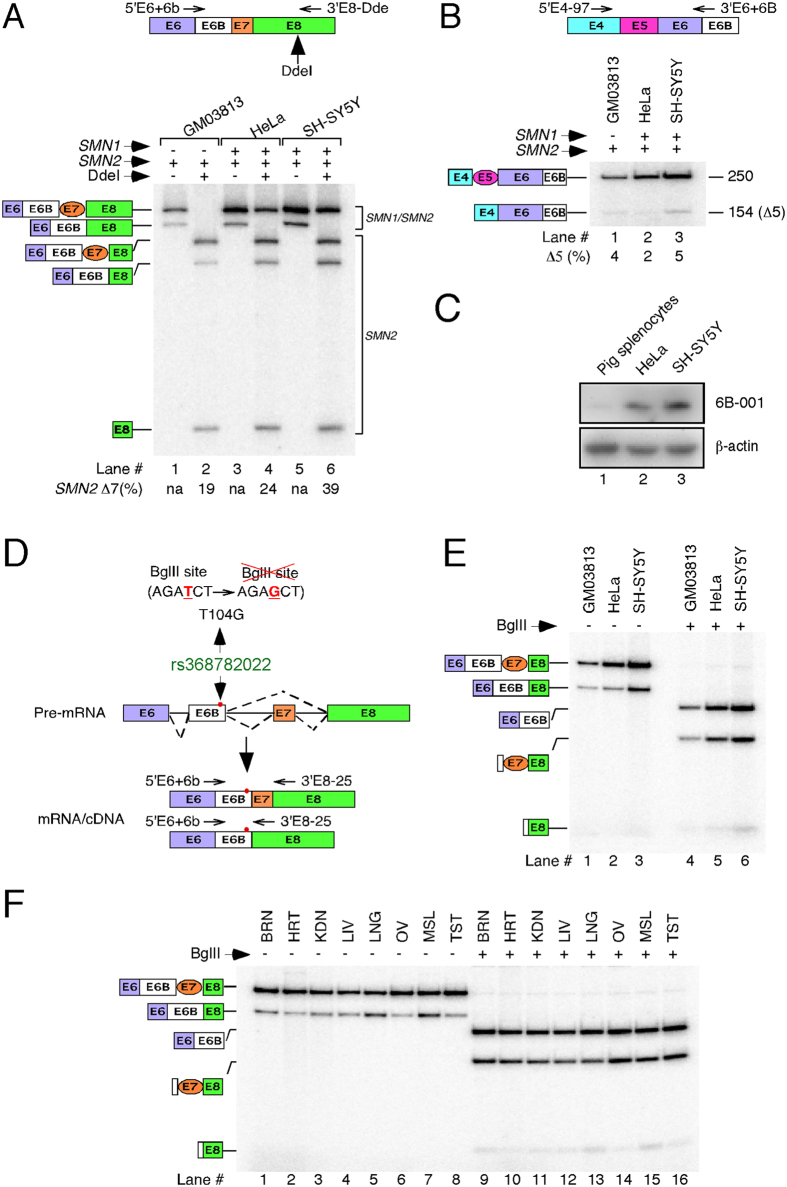
Both *SMN* genes include exon 6B. **(A)** *SMN1* and *SMN2* transcripts were distinguished by RT-PCR followed by DdeI digestion similarly as in Singh *et al.*^31^. Annealing positions of primers used for PCR and DdeI restriction site are given in the top panel. Spliced isoforms are indicated on the left of the gel. The percentage of *SMN2* exon 7 skipping in DdeI-digested samples is indicated. na: not applicable. Of note, PCR amplification described is meant to analyze only those transcripts that include exon 6B. Majority of SMN transcripts do not include exon 6B and will not be amplified by this approach. **(B)** Exon 6B-containing *SMN* transcripts in which exon 5 was either included or skipped were amplified by RT-PCR. Other descriptions are the same as in (**A**). **(C)** Western blot showing expression of SMN6B protein using exon 6B-specific antibody 6B-001. β-actin served as a loading control. **(D)** Diagrammatic representation of rs368782022 allelic variation (T104G substitution) that abrogates BglII site. BglII site and annealing positions of primers used for amplification of *SMN* exon 6B-containing transcripts are shown. **(E)** Probing of *SMN* exon 6B for rs368782022 allelic variation in GM03813, HeLa and SH-SY5Y cells. BglII endonuclease cleaved PCR amplified products, confirming the lack of T104G substitution in transcripts of all cell types examined. Spliced isoforms are indicated on the left of the gel. **(F)** Probing of *SMN* exon 6B for rs368782022 allelic variation in various human tissues examined in Fig. 1D. BglII endonuclease cleaved PCR amplified products, confirming the lack of T104G substitution in all human tissues examined. Spliced isoforms are indicated on the left of the gel. Tissue abbreviations are the same as indicated in Fig. 1B.

To determine whether both *SMN* genes are capable of expressing exon 6B-containing transcripts, we employed three cell lines (cultured cells): type I SMA patient fibroblasts (GM03813), neuronal SH-SY5Y and non-neuronal HeLa cells. While GM03813 contains only *SMN2*, SH-SY5Y and HeLa cells contain both *SMN1* and *SMN2*. Due to low abundance, we were unable to detect appreciable levels of exon 6B-containing transcripts in cultured cells employing MESDA. Hence, we adopted a modified protocol in which we used forward and reverse primers annealing to exon 6/exon 6B junction and exon 8, respectively (Fig. 2A). Taking advantage of the *SMN2*-specific DdeI restriction site^12^, we confirmed the expression of 6B-containing transcripts harboring exon 7 (*SMN6B*) from both *SMN* genes (Fig. 2A). However, transcripts that contained exon 6B but lacked exon 7 (*SMN6BΔ7*) were produced only from *SMN2. SMN6B* constituted an overwhelming majority (>75%) of exon 6B-containing *SMN2* transcripts generated in GM03813 and HeLa cells, suggesting a stimulatory effect of exon 6B inclusion on splicing of exon 7. In the case of neuronal SH-SY5Y cells, we observed a ~2-fold increase in *SMN6BΔ7* compared to those observed in GM03813 and HeLa cells. Levels of *SMN6B* in SH-SY5Y cells were about ~1.5-fold higher than *SMN6BΔ7*.

Considering exon 5 skipping is a detectable event for both *SMN* genes^31^, we wanted to assess the levels of *SMN6BΔ5* transcripts that harbor exon 6B but lack exon 5. For this, we performed PCR using forward and reverse primers that annealed to exon 4 and exon 6/exon 6B junction, respectively. We detected very low levels of *SMN6BΔ5* transcripts, suggesting skipping of exon 5 with inclusion of exon 6B is a rare combination of events (Fig. 2B). These results are consistent with the observations in allele C mice, where *SMN6B* transcript was the most prevalent exon 6B-containing isoform (Fig. 1B). To test the expression of endogenous SMN6B protein, we raised mouse polyclonal antibodies (6B-001) against a unique polypeptide encoded by exon 6B. Despite low expression, we expected detection of endogenous SMN6B in HeLa and SH-SY5Y cells, since they carry both *SMN1* and *SMN2* that generate *SMN6B*. Indeed, Western blot results confirmed the endogenous expression of SMN6B in these cell lines (Fig. 2C). However, due to poor reactivity of 6B-001 coupled with low expression of exon 6B-containing transcripts in GM03813 cells, we could not detect SMN6B in GM03813 cells (not shown).

An allelic polymorphism substituting T with G residue at the 104^th^ position (T104G) of exon 6B has been reported (rs368782022) (Fig. 2D). Since T104G mutation is located after a stop codon, it does not change the amino acid sequence coded by exon 6B. However, this mutation may affect splicing of exon 6B by abrogating and/or creating cis-regulatory element(s). To determine whether this polymorphism is present within transcripts generated from human cell lines and tissues used in our study, we took advantage of a BglII endonuclease site abrogated by T104G mutation (Fig. 2D). We amplified the exon 6B-containing transcripts using forward and reverse primers annealing to exon 6/exon 6B junction and exon 8, respectively. We observed complete cleavage of these PCR products by BglII, confirming the absence of the rs368782022 allele in all cell lines and tissues examined in this study (Fig. 2E,F).

### Exon 6B is derived from a primate-specific Alu-like sequence

**Figure 3:**
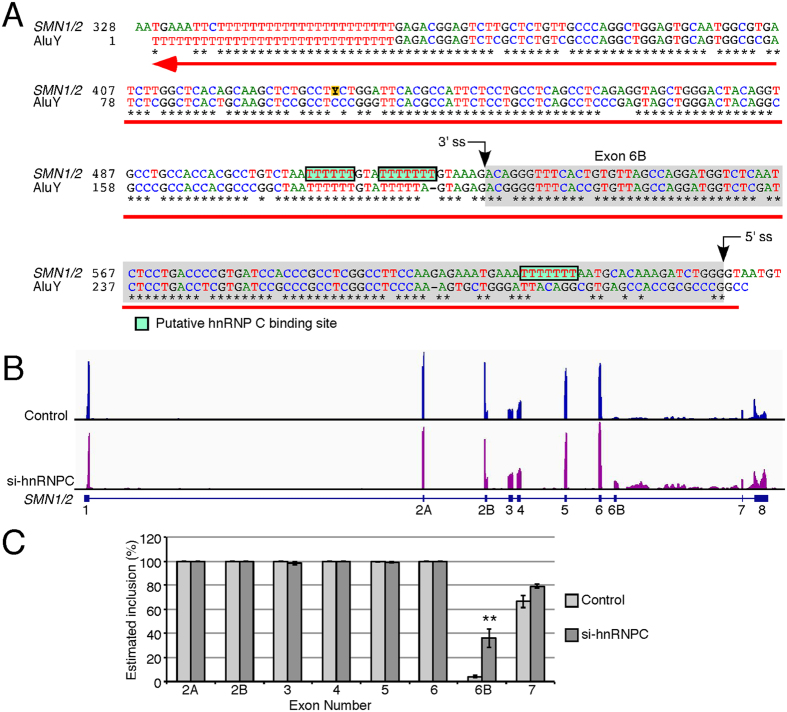
Exon 6B is derived from an intronic Alu element. **(A)** Alignment of *SMN* intron 6 region spanning exon 6B. Numbering starts from the beginning of intron 6. Exon 6B and putative binding sites for hnRNP C are highlighted in grey and green, respectively. Black arrows indicate splice site (ss) positions and red arrow indicates position and direction of Alu insertion. AluY sequences (reverse and complement) are obtained from Dfam (Accession: DF0000002). **(B)** Genomic view of RNA-Seq of HeLa cells with and without hnRNP C depletion as previously described by Zarnack *et al.*^35^. Exon positions are shown at the bottom. **(C)** Estimated exon inclusion for all internal exons of *SMN* based on sequencing data from (**B**). Error bars represent standard error of 4 sequencing libraries. Statistical significance: **p < 0.01.

To determine the evolutionary origin of *SMN* exon 6B, we analyzed the entire *SMN* gene for transposable elements. We observed an abundance of Alu-like sequences within introns of *SMN* gene (Supplementary Fig. 2). A typical Alu element is ~300 nt long and consists of two similar monomers: the left and right arms separated by an A-rich sequence and followed by a poly-A tail^33^. Although the antisense sequence of the right arm of an intronic Alu element is associated with the most reported exonization events, either arm or the antisense sequence of the entire Alu element could be exonized^4^,^34^. Interestingly, exon 6B mapped to the left arm of the antisense sequence of an Alu element that was found to be present in all members of Hominidae family (Fig. 3A and Supplementary Fig. 3A). Supporting the low abundance of exon 6B-containing transcripts, the predicted strengths of splice sites of exon 6B were substantially lower than that for the neighboring exons 6 and 7 (Supplementary Fig. 3B).

The antisense strand of an Alu element is often utilized for an exonization event due to the presence of several splice site (ss)-like motifs/signal, including a polyuridine (polyU) tract. PolyU provides the binding site for U2AF65 that defines the 3′ ss of an exon. A recent transcriptome-wide analysis has shown that hnRNP C competes with U2AF65 for interaction with PolyU at the putative 3′ ss of an Alu-like exon and protects the transcriptome from exonization of Alu elements^35^. We analyzed the publicly available RNA-Seq data from this study by mapping reads to *SMN* pre-mRNA in hnRNP C-depleted and control samples (Fig. 3B). In hnRNP C-depleted samples, the height of the peak corresponding to exon 6B, as well as the proportion of junction-spanning reads containing sequences derived from exon 6B, showed more than 10-fold increase (Fig. 3B,C). A fine analysis of mapped sequences confirmed the accurate boundary of exon 6B. These findings independently confirmed the exonization of exon 6B and suggested a role for hnRNP C in regulation of exon 6B splicing. We performed similar analysis of publicly available RNA-Seq data from cells depleted of several other hnRNPs and pyrimidine-binding proteins. None of the examined factors including hnRNP A1, hnRNP A2/B1, hnRNP F, hnRNP H1, hnRNP M, hnRNP U, PTB1, HuR, and TIA1/TIAR showed any appreciable effect on splicing of *SMN* exon 6B (Supplementary Fig. 4).

### Exon 6B-containing transcripts are substrates of NMD

**Figure 4:**
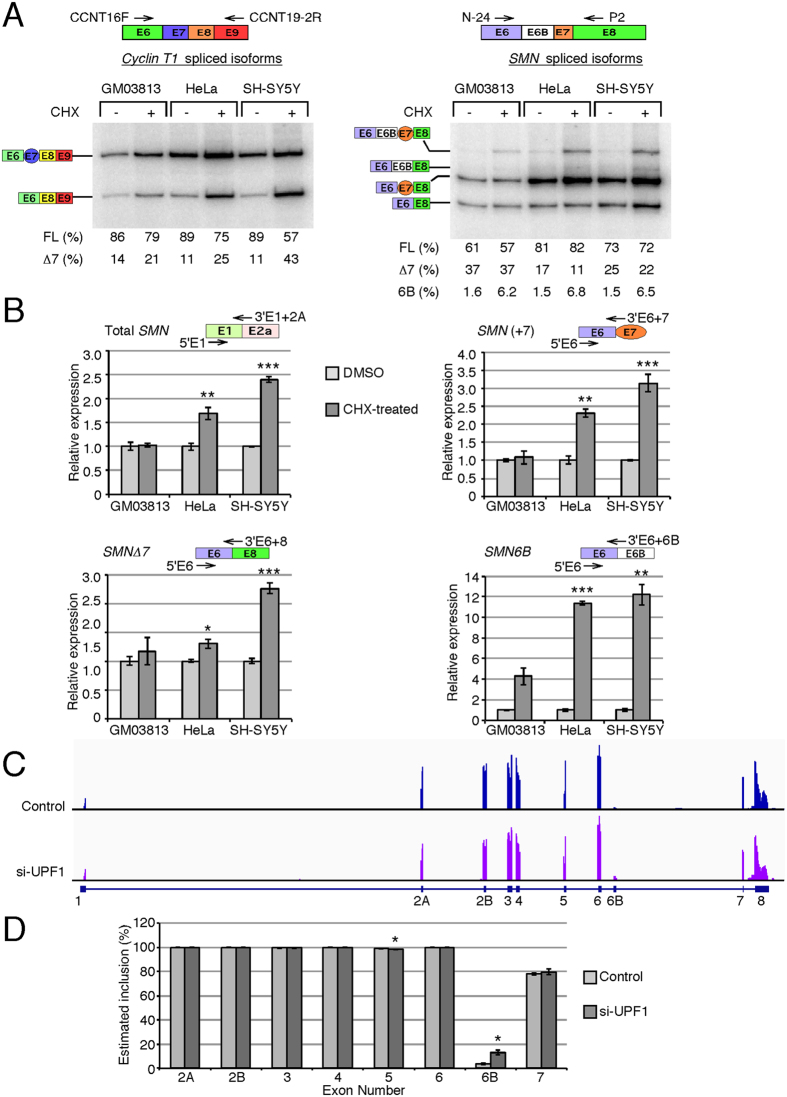
Susceptibility of exon 6B-containing transcripts to NMD. **(A)** RT-PCR results for *Cyclin T1* and *SMN* transcripts are shown in the left and the right panels, respectively. Cells were treated with DMSO (−) or 20 μg/ml CHX (+) for 6 h. Other descriptions are the same as in Fig. 2A. FL: full-length. **(B)** Quantification of various *SMN* transcripts by QPCR under CHX treatment. The identity of each isoform and annealing position of primers used to amplify them are indicated above each graph. Values are expressed as relative to untreated samples. Error bars represent the standard error of 3 biological replicates. **(C)** Genomic view of RNA-Seq of HeLa cells with and without UPF1 depletion (SRA accession number SRP063462). Exon positions are shown at the bottom. **(D)** Estimated exon inclusion for all internal exons of *SMN* based on sequencing data from (**C**). Error bars represent standard error of 2 sequencing libraries. Statistical significance: *p < 0.05, **p < 0.01, ***p < 0.001.

Inclusion of exon 6B creates a PTC after the 16^th^ amino acid and makes *SMN6B* transcript a likely substrate of NMD (Fig. 1C). NMD is a translation-dependent process and inhibition of translation has been known to increase the stability of the PTC-bearing transcripts^9^. To confirm that *SMN6B* transcripts are prone to NMD, we treated SMA patient cells (GM03813), HeLa and neuronal SH-SY5Y cells with cycloheximide (CHX; a translation inhibitor) and determined *SMN* transcript levels by either splicing assays employing forward and reverse primers in exons 6 and 8, respectively, or by QPCR using exon-specific forward and junction-specific reverse primers (Fig. 4). As a treatment control we examined levels of *Cyclin T1Δ7* isoform, a bona fide substrate of NMD^36^. *Cyclin T1* showed the expected increase in transcripts without exon 7 when translation was inhibited (Fig. 4A). In case of *SMN*, we recorded a ~4-fold increase in the levels of *SMN6B* (in reference to *SMN1*; GenBank: KU524731 and to *SMN2*; GenBank: KU524733) and a small but detectable increase in the levels of *SMN6BΔ7* (referred to *SMN2*; GenBank: KU524732) in CHX-treated cells (Fig. 4A). In the QPCR assay, we observed a ~10-fold increase in the levels of exon 6B-containing transcripts in CHX-treated cells (Fig. 4B). These observations support that exon 6B-containing transcripts are NMD substrates. Interestingly, we observed a general increase in the levels of *SMN* transcripts in HeLa and SH-SY5Y cells upon repression of translation (Fig. 4B). This observation is not totally surprising, since natural transcripts are also subjected to NMD, particularly the ones with long 3′ untranslated regions (3′UTR)^9^. However, the general increase in *SMN* transcripts in CHX-treated cells was substantially lower than the increase in the exon 6B-containing transcripts, suggesting that exon 6B-containing transcripts are bona fide substrates of NMD.

Among multiple factors involved in regulation of NMD, upframeshift protein 1 (UPF1) plays a key role^37^. To furtherer validate that the exon 6B-containing transcripts are substrates of NMD, we analyzed publicly available RNA-Seq data from UPF1-depleted HeLa cells (NCBI/SRA accession number SRP063462). We mapped sequence reads to the entire *SMN* pre-mRNA. Compared to control cells, we observed >4-fold increase in peak height corresponding to *SMN* exon 6B and junction-spanning reads containing exon 6B sequences in UPF1-depleted cells (Fig. 4C,D). There was no change in expression of other *SMN* exons except for a very small but significant decrease in inclusion of exon 5. These findings independently confirmed that the exon 6B-containing transcripts are substrates of NMD.

### SMN6B has intermediate stability

**Figure 5:**
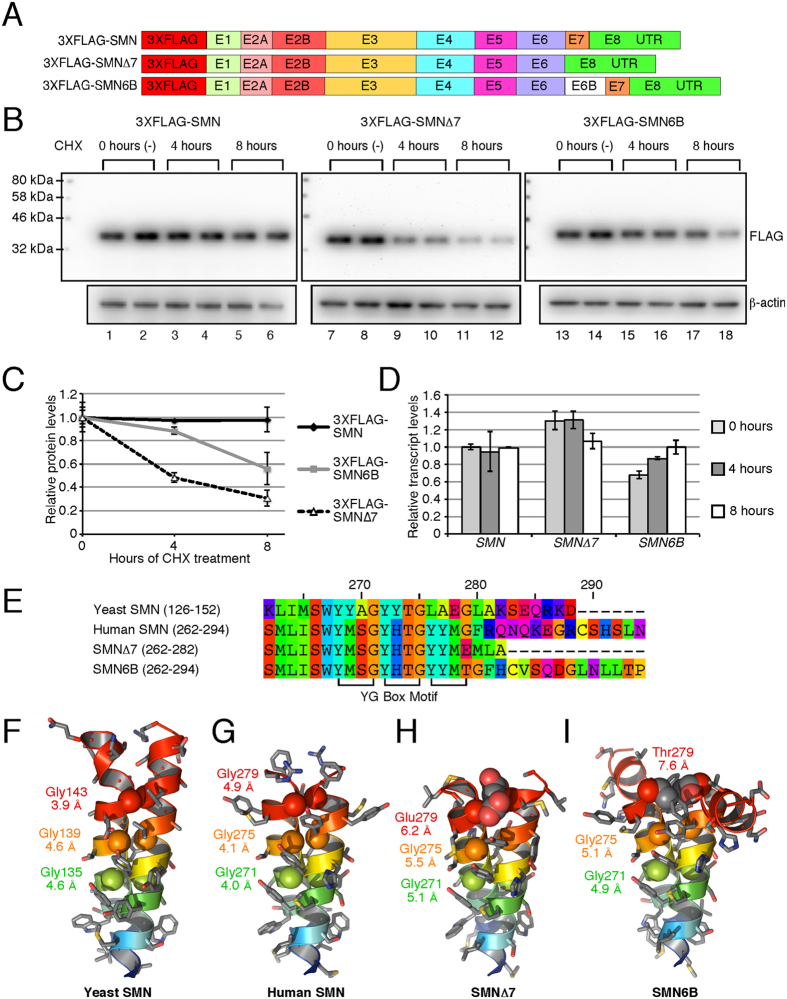
Stability of SMN6B protein. **(A)** Diagrammatic representation of the cDNA constructs used for the determination of protein stability. **(B)** Western blots showing the effect of 20 μg/ml CHX treatment on the level of 3XFLAG-tagged proteins. 12 μg of total protein was loaded in each lane. **(C)** Densitometric quantification of Western blots shown in (**B**). Values are expressed as relative to the average value at 0 hours. Error bars represent standard error. **(D)** QPCR of transfected *SMN* after CHX treatment. We generated cDNA employing 3′UTR primer. QPCR were performed using primers 5′FLAG (anneals within 3XFLAG) and 3′E1+2A (anneals exon 1/exon 2A junction). All values are expressed as relative to untreated SMN and normalized by neomycin resistance gene on the same transfected plasmid using primers 5′NEO (anneals within neomycin) and 3′RT-Univ (anneals 3′UTR adapter). Error bars represent standard error. **(E)** The sequence alignment compares the YG Box domains of *Schizosaccharomyces pombe* (yeast) SMN (UniProtKB accession: Q09808), human SMN (UniProtKB accession: Q16637-1), SMN∆7 (UniProtKB accession: Q16637-3) and SMN6B proteins. Residue reference numbers at the top are based on the human SMN. The positions of the conserved YG Box motif comprised of the (YXXG)_3_ tetrad repeat sequence is indicated at the bottom. **(F,G)** The YG Box domains mediate dimerization through a coiled-coil interaction observed in the crystal structure of the yeast SMN (PDB code: 4RG5) and human SMN (PDB code: 4GLI) proteins. **(H,I)** Symmetric homology modeling of the YG Box domains from the SMN∆7 and SMN6B isoforms shows the potential for steric distortion in the dimeric structures due to substitution of the last conserved residue of the YG Box motif, Gly279. Structure and models are shown in cartoon representations with a blue to red rainbow color scheme from N- to C-terminus. The last six residues in the SMN6B model are unstructured and not shown for clarity. Side chains are shown as stick representations with coloring by element: grey for carbon, red for oxygen, blue for nitrogen, and gold for sulfur. The Cα atoms of the conserved glycine residues are shown as van der Waals spheres. The inter-atomic distances between Cα atoms of the symmetry related glycine residues are indicated below the labels for each residue.

To compare the stability of SMN, SMNΔ7 and SMN6B proteins, we constructed mammalian expression vectors harboring the complete human coding sequences (Fig. 5A). We included the entire 3′UTR in our expressed transcripts to preserve the unique binding sites of microRNAs that might affect protein expression. For detection purposes, we added a 3XFLAG tag at the N-terminus of these proteins. We transfected HeLa cells with these expression vectors and allowed the cells to accumulate sufficient levels of expressed proteins. We then treated cells with CHX to stop translation and determined the levels of residual proteins at 4 and 8 h post CHX treatment. As expected, levels of SMN remained the same relative to the β-actin loading control during the entire 8 h of CHX treatment, whereas levels of SMNΔ7 and SMN6B declined during this time (Fig. 5B,C). However, the residual SMN6B level was noticeably higher than SMNΔ7 level even after 8 h of CHX treatment. We performed QPCR to rule out the possibility that the results were impacted by transcript levels (Fig. 5D). A previous report has shown that SMN is >3-fold more stable than SMNΔ7 28. Our results are consistent with those observations. They also indicate that SMN6B is ~2-fold less stable than SMN, but ~2-fold more stable than SMNΔ7.

A model system using maltose binding protein (MBP) fused to the N-terminus of the YG domain (MBP-YG) showed that oligomerization of SMN is mediated by a core YG dimer^29^. In structures of both the yeast and human YG domains (Fig. 5F,G), the YG dimer is formed through a non-heptad repeat coiled-coil interaction^26^,^29^. The sequence of the YG domain is defined by three tetrad repeats of a conserved YG box motif (YXXG)_3_, which overlaps a tetrad repeat pattern of serine and threonine residues (Fig. 5E). The dimeric structure of the YG domain is similar to the glycine zipper (Supplementary Fig. 5), which is a common motif that mediates interactions between transmembrane helices^38^. In both SMNΔ7 and SMN6B proteins, the last glycine in the YG box motif (Gly279) has been replaced with either glutamate or threonine (Fig. 5E). Comparative modeling of the YG domains of SMNΔ7 (Fig. 5H) and SMN6B (Fig. 5I) shows that the helices in both models are splayed apart with increased distances between the Cα atoms of the symmetrical glycine residues. Accommodation of either a charged Glu279 in SMNΔ7 or a bulky beta-branched Thr279 in SMN6B causes an increase in the inter-helical distance. In the MBP-YG model system, a single amino acid substitution of Gly279 with a glutamate residue is sufficient to prevent oligomerization^29^. Disruption of the coiled-coil interaction in the YG domain, as seen in the comparative models of both SMNΔ7 and SMN6B, potentially decreases oligomerization leading to increased turnover of these proteins.

### SMN6B interacts with key regulator Gemin2

**Figure 6:**
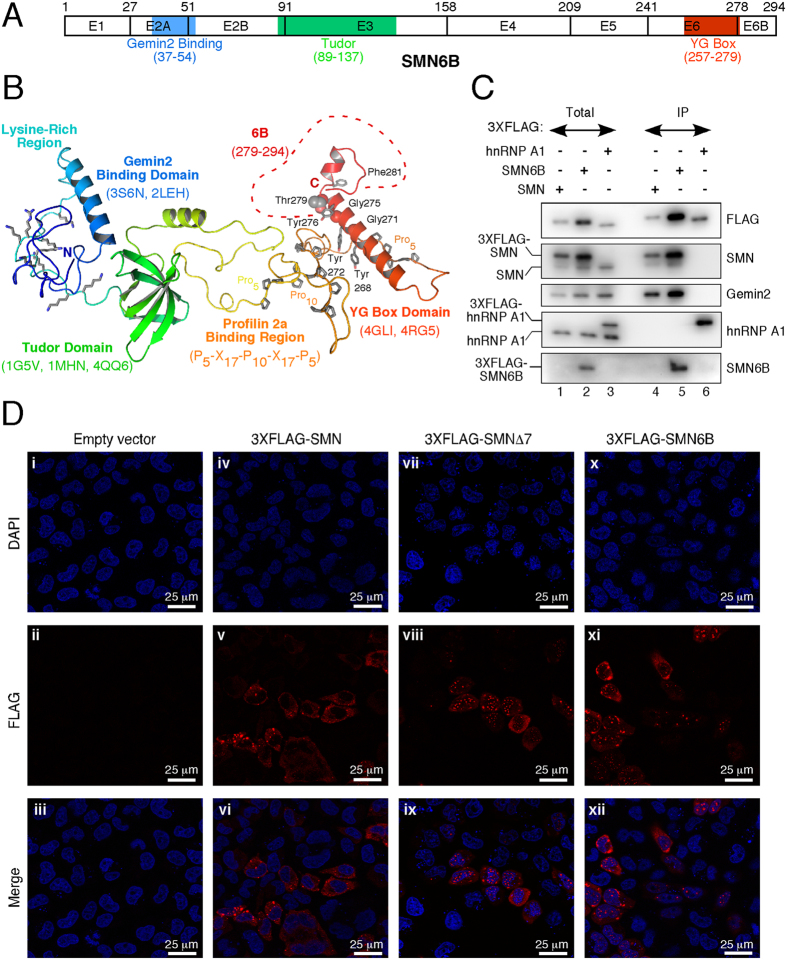
Interacting partners and subcellular localization of SMN6B. **(A)** Diagrammatic representation of the functional domains of SMN6B. Exons are shown as boxes with the number of last amino acid residue indicated above. Gemin2 binding, Tudor, and YG domains are highlighted in different colors. **(B)** The model of the SMN6B protein was calculated based on multiple structure templates using comparative modeling. The SMN6B model is shown as a cartoon representation with a blue to red rainbow color scheme from N- to C-terminus. The structured domains are indicated with the PDB codes of the comparative modeling templates listed below each name. The unstructured regions highlighted include the lysine-rich region, and the Profilin 2a binding region with the conserved proline-rich sequence indicated below the label. The region derived from exon 6B is denoted by a red dashed line and includes residues 279–294 as indicated in the label. The conserved residues of the YG Box motif are indicated with tyrosine residues shown in stick representations and the Cα atoms of the glycine residues shown as van der Waals spheres. The variant residue Thr279 is also indicated with side chain heavy atoms shown as spheres. **(C)** SMN6B interacting proteins. HeLa cells transfected with expression vectors for 3XFLAG-SMN, 3XFLAG-SMN6B, or 3XFLAG-hnRNP A1 were used to prepare whole-cell extracts. SMN protein complexes were then pulled down by immunoprecipitation (IP) using anti-FLAG magnetic beads, eluted with the FLAG peptide, and analyzed by Western blotting. Bands corresponding to the FLAG-tagged proteins as well as their endogenous counterparts are indicated on the left of each panel, antibodies used are indicated on the right. Total represents 10% of the extract used in the IP. **(D)** Subcellular localization of SMN protein isoforms. HeLa cell were transfected with either an empty vector (control) or with the expression vectors for the SMN constructs indicated on the top of the corresponding panels. ~24 hours later cells were processed and subcellular distribution of 3XFLAG-tagged SMN proteins was examined by immunocytochemistry using anti-FLAG antibody conjugated to Alexa 555 (red). Nuclei were stained with DAPI (blue). Scale bar is 25 μm.

The SMN-Gemin2 interaction has been linked to several SMN functions, including snRNP biogenesis, signal recognition particle biogenesis, DNA recombination, and translation regulation^15^,^17^,^18^,^19^. Moreover, SMN co-localizes with Gemin2 in motor neuron axons indicating that this complex has a role in motor neuron trafficking of mRNAs^24^. The structured domains of SMN correlate closely with the exon boundaries, such that the Gemin2 binding domain is found mostly within exon 2A, the Tudor domain is associated with exon 3 and most of the YG domain is contained within exon 6 (Fig. 6A). Comparative modeling of the FL SMN6B protein predicts three structural domains that are independently folded and do not interact with each other (Fig. 6B). The inclusion of exon 6B is predicted to disrupt the C-terminus of the helix in the YG domain with a break in the helix immediately following the variant Thr279, followed by a short extension of the helix to Asp287 (Fig. 6B). In the crystal structure of the YG domain of SMN, the helix ends at Phe280 and the C-terminal region from residues 282–294 is disordered^29^. These observations support that an ordered C-terminus is not required for the overall structural integrity of SMN and SMN6B. These observations also suggest that SMN6B, similar to SMN, will retain the ability to interact with Gemin2.

To test whether SMN6B interacts with Gemin2, we transfected HeLa cells with 3XFLAG-SMN6B. As a positive control, we performed a parallel experiment with 3XFLAG-SMN. As a negative control, we included a side-by-side experiment with 3XFLAG-hnRNP A1, which does not interact with either SMN or Gemin2. Total cell lysates prepared from the transfected cells were used for immunoprecipitation with anti-FLAG antibodies, and bound proteins were analyzed by Western blotting. Supporting that SMN6B is functionally equivalent to SMN, both 3XFLAG-SMN and 3XFLAG-SMN6B pulled down Gemin2 (Fig. 6C). Underscoring the specificity of interaction between SMN/SMN6B with Gemin2, 3XFLAG-hnRNP A1 did not pull down Gemin2. 3XFLAG-SMN6B also pulled down endogenous SMN, supporting formation of heterodimeric complexes between these two proteins. Due to low expression of endogenous SMN6B and poor reactivity of antibody against SMN6B, we were unable to detect SMN6B pulled down by 3XFLAG-SMN.

Previous studies have established the critical role of the C-terminal amino acids of SMN in its subcellular distribution. In particular, removal of the exon 7-coding sequence has been shown to prevent export of SMN from the nucleus and promote nuclear granule formation, a highly regulated process for the viability of cells^39^,^40^. It has been also shown that the exon 7-coding sequence facilitates cytoplasmic stress granule formation^39^. We performed confocal microscopy to monitor the subcellular distribution of 3XFLAG-tagged SMN6B, SMN and SMNΔ7 proteins expressed in HeLa cells. As expected, we observed predominant localization of overexpressed SMN and SMNΔ7 in cytoplasmic and nuclear granules, respectively (Fig. 6D). We also observed non-granular (diffused) localization of both proteins in the cytosol, although the intensity of such distribution was less pronounced. Localization of 3XFLAG-SMN6B showed an intermediate pattern as evidenced by the presence of high and low counts of cytosolic and nuclear granules, respectively (Fig. 6D). Similar to SMN, overexpressed SMN6B also showed diffused cytoplasmic localization. The outcome of our localization study should be interpreted with caution, since a similar level of expression of SMN isoforms through transient transfections could not be maintained. However, our findings suggest that SMN6B may retain most of the nuclear and cytosolic functions of SMN.

## Discussion

Here we report a novel splicing event, triggered by exonization of an intronic Alu element of human *SMN* genes. We call this novel exon 6B, since it is incorporated after the constitutively spliced exon 6. The serendipitous discovery of exon 6B was facilitated by MESDA, an unbiased method that accurately captures the relative abundance of various splice variants of *SMN* in a single reaction. We detected exon 6B-containing transcript in all tissues of allele C mice. We subsequently validated the expression of exon 6B-containing transcripts in human tissues and cell lines. We confirmed that the inclusion of exon 6B occurs in both *SMN1* and *SMN2.* The majority of exon 6B-containing transcripts were *SMN6B* that harbored exon 7. The next most abundant exon 6B-containing transcript was *SMN6BΔ7* generated from *SMN2*. Both *SMN6B* and *SMN6BΔ7* code for the same SMN6B protein. Despite low abundance of *SMN6B* and *SMN6BΔ7*, we confirmed the expression of endogenous SMN6B protein in both neuronal and non-neuronal cells.

Although an overwhelming 39% of *SMN* sequence is occupied by ~40 Alu-like sequences, exon 6B represents the first known exonization event of an Alu element within *SMN*. In contrast to the right arm of an antisense sequence of an Alu being the most prevalent source of exons/exonization^4^, exon 6B originated from the left antisense arm of an Alu element. Consistent with a new hypothesis that hnRNP C is a universal suppressor of exonization of Alu-like sequences^35^, we confirmed enhanced expression of exon 6B-containing transcripts in hnRNP C-depleted samples, using publicly available RNA-Seq data. Interestingly, hnRNP C was recently shown to be a novel component of stress granules^41^. Hence, either conditions of stress granule formation that might limit the availability of free hnRNP C or downregulation of expression of hnRNP C are likely to favor inclusion of exon 6B in *SMN* transcripts. A recent comparison of CLIP-Seq data of 51 human proteins revealed a much larger spectrum of RNA-protein interactions involving transcripts derived from transposable elements including Alu elements^42^. The findings of this study suggest a diversity of mechanisms by which transcripts harboring Alu-derived exons are likely to be generated and stabilized. Our analyses of publicly available RNA-Seq data from splicing factor-depleted cells did not capture a significant effect of hnRNP A1, hnRNP A2/B1, hnRNP F, hnRNP H1, hnRNP M, hnRNP U, PTB1, HuR, and TIA1/TIAR on splicing of *SMN* exon 6B (Supplementary Fig. 4). However, RNA-Seq data should be interpreted with caution, since the results could be impacted by number of biological replicates analyzed, growth conditions, degree and duration of depletion and the type of cells used.

Inhibition of translation substantially increased *SMN6B* and *SMN6BΔ7* levels, confirming that exon 6B-containing transcripts are bona fide substrates of NMD (Fig. 4). Analysis of publicly available RNA-Seq data from UPF1-depleted HeLa cells independently validated the NMD-mediated downregulation of exon 6B-containing transcripts (Fig. 4C,D). NMD is inhibited by various cellular stresses, including infection, nutrient deprivation, reactive oxygen species, oxygen deprivation (hypoxia) and double-stranded RNA^10^. Hence, expression of exon 6B-containing transcripts is likely to be higher under stress-associated conditions. Interestingly, a recent report provides an example of local inhibition of NMD at axonal terminals so that NMD-sensitive *Robo3.2* transcripts (GenBank: NM_001164767) are translated, as Robo3.2 protein isoform is essential for the navigational functions of growth cones^43^. A similar translational regulation of exon 6B-containing transcripts cannot be ruled out, given the critical role of SMN in axon outgrowth and cytoskeletal dynamics.

Replacement of sixteen residues of SMN (GF**R**QNQ**KE**G**R**CSHSLN; charged and hydrophobic residues are in bold and underlined, respectively) with sixteen residues of SMN6B (TGFHCVSQ**D**GLNLLTP) at the C-terminus results in the gain of four hydrophobic and loss of three charged residues. We have captured the effect of these changes on protein stability. SMN6B displayed intermediate stability compared to SMN and SMNΔ7 (Fig. 5B,C). A previous study linked very low stability of SMNΔ7 to a potential degradation signal comprised of the truncated YG domain and the four C-terminal amino acid residues, EMLA, encoded by exon 8 28. The longer C-terminal region of SMN6B could be one of the driving forces for its higher stability compared to SMNΔ7. Stability of SMN is linked to oligomerization that requires formation of a core YG dimer^29^. A comparison of structures of the C-terminus generated by comparative modeling showed disrupted coiled-coil interactions in the YG domains of SMNΔ7 and SMN6B (Fig. 5F–I). This disruption is likely to be less pronounced for a heterodimer formed between SMN and SMB6B monomers. Formation of this heterodimer may contribute at least in part towards the intermediate stability of SMN6B.

SMN tightly interacts with Gemin2, and formation of the SMN-Gemin2 core complex is critical for all known functions of SMN. Considering that SMN6B and SMN share several critical domains, our finding that SMN6B fully retains the capability to interact with Gemin2 suggests overlapping functions between SMN6B and SMN (Fig. 6C). However, a dramatic change in the composition of its C-terminus has implications for novel functions of SMN6B. A previous study has suggested the presence of a nuclear export signal in exon 7 based on the fact that the removal of the exon 7-coding sequence caused SMN retention in the nucleus^27^. Accordingly, transient overexpression of SMN predominantly localizes SMN to cytoplasmic granules. Cytoplasmic localization of SMN is consistent with the motor neuron-specific role of SMN in trafficking of cargos across the long processes of axons. Notably, overexpression of SMN6B did not restrict its distribution to either the nucleus or to cytoplasmic granules (Fig. 6D). Also, we did not observe specific targeting of SMN6B to membranes, despite its hydrophobic C-terminus. Rather, SMN6B was distributed across the cytosol and the nucleus. These results suggest that SMN6B is readily available for its function even under stress-associated conditions, when SMN is trapped within stress granules.

Expression of SMN6B is expected to be higher in individuals with high copy numbers of *SMN1* and/or *SMN2*. Our results reveal that the inclusion of exon 6B has a suppressive effect on *SMN2* exon 7 skipping (Fig. 2A). Based on the fact that high *SMN2* copy number reduces the severity of SMA, SMN6B may contribute in part towards reducing the severity of the disease in these patients. Genetic mutations and/or availability of splicing factors that regulate the expression of SMN6B would likely modulate the severity of the disease. Evidence is emerging that intergenic Alu-like sequences could potentially alter the C-terminus of several proteins^44^. While the significance of such changes is not always clear, the consequences could be drastic in cases where the C-terminus plays an essential role. Interestingly, the EST database revealed several transcripts coding for proteins with a C-terminus almost identical to the one encoded by *SMN* exon 6B (Supplementary Fig. 6). However, the expression and functional significance of proteins encoded by these transcripts remain to be investigated. Now that we have uncovered SMN6B, the stage is set for the exploration of unique functions of SMN6B and other proteins harboring similar C-termini.

## Materials and Methods

All methods were performed in accordance with the approved biosafety and radiation safety guidelines of Iowa State University (ISU), adhering to the federal and state guidelines. All animal experiments were carried out in accordance with the approved protocols by the Institutional Animal Care and Use Committee (IACUC) of ISU, adhering to the guidelines of American Veterinary Medical Association (AVMA), United States Health and Human Services (US HHS), United States Department of Agriculture (USDA) and State of Iowa.

### Mice

Allele C mice with C57BL/6J background (*Smn*^C/C^, C is defined by a chimeric gene plus *SMN2*), were generated from breeding pairs obtained from Jackson Laboratory (stock number 008714)^32^ and genotyped by ear punch. The mice were housed in standard conditions: constant temperature (22 ± 1 °C), humidity (relative, 30%) and a 12 h light/dark cycle, where they were monitored daily for health. All mice had free access to food and water. Six to eight week old mice were anaesthetized using isoflurane and then sacrificed by means of cervical dislocation. Tissues including brain, heart, kidney, liver, lung, muscle, spinal cord, uterus/ovary and testis were dissected out and flash-frozen immediately in liquid nitrogen or dry ice and stored at −80 °C.

### Cell culture and treatment

Unless otherwise noted, all tissue culture media, supplies, and transfection reagents were purchased from Life Technologies. Cell lines used are previously described^31^. For transfection experiments, cells were transfected with X-tremeGENE HP (Roche) or Lipofectamine 2000 (Life Technologies) following the manufacturer’s instructions. CHX was obtained from Sigma. More detailed methods can be found in Supplementary Experimental Methods.

### RT-PCR

Total RNA was isolated using TRIzol reagent (Life Technologies) and treated with RQ1 RNase-free DNase (Promega). RNA isolated from human tissue was purchased from Ambion (FirstChoice Human Total RNA Survey panel); samples from each tissue were pooled from three individuals. cDNA was generated using SuperScript III (Life Technologies) and amplified using Taq DNA Polymerase (New England Biolabs) in the presence of either a 5′-end-^32^P-labelled primer or a trace amount of [α-^32^P] dATP (3,000 Ci/mmole; Perkin-Elmer). MESDA was performed as previously described^31^. For quantitative real-time PCR (QPCR), reactions were carried out using FastStart Universal SYBR Green Master Mix (Roche). For detailed methods, see Supplementary Experimental Methods. All primer sequences appear in Supplementary Table 1.

### Identification of SMN spliced isoforms

PCR was carried out for 30–35 cycles using Taq DNA polymerase (New England Biolabs) and cDNA produced from allele C mouse brain total RNA. Bands corresponding to products of interest were gel purified and cloned into the pGEM-T easy vector following the manufacturer’s recommendations (Promega). Randomly selected positive colonies were propagated and used to purify plasmid DNA employing QIAprepSpin Miniprep Kit (Qiagen). Purified plasmids were then sequenced at the DNA facility of Iowa State University.

### Generation of polyclonal antibodies (anti-6B-001)

A 6B polypeptide (C-TGFHCVSQDGLNLLTP) was synthesized at the protein core facility of Iowa State University. Antibodies were generated at the Hybridoma facility of Iowa State University. Briefly, 6B peptide was conjugated to KLH using Imject^TM^ Maleimide-Activated mcKLH Spin Kit (Thermo Scientific) and 25 μg used for 4 intraperitoneal injections on a 14-day schedule. Immunoglobulin (IgG) was purified from the polyclonal fluids with Protein A column (Pierce). More detailed methods can be found in Supplementary Experimental Methods.

### Plasmid constructs

3XFLAG-SMN, 3XFLAG-SMN6B, and 3XFLAG-SMNΔ7 mammalian expression vectors were generated as follows. Human *SMN*, *SMN6B*, and *SMNΔ7* sequences were amplified by PCR with Phusion DNA polymerase (New England Biolabs) using as a template cDNA prepared from total RNA isolated from allele C mouse brain. The amplified sequences were then cloned in the MluI-SalI restriction sites of the 3XFLAG-hTIA1 mammalian expression vector^45^. All vectors were sequenced before being used. All restriction enzymes and Quick Ligation Kit were from New England Biolabs.

### Western blot analysis

Whole-cell lysates from HeLa and SH-SY5Y were prepared using ice-cold radioimmunoprecipitation assay (RIPA) buffer (Boston BioProducts; See Supplementary Experimental Procedures). Protein samples were resolved on 11 or 12% SDS-polyacrylamide gels, transferred to membranes (Immun-Blot PVDF Membrane for Protein Blotting, Bio-Rad) using Transfer-Blot Turbo Transfer System (Bio-Rad), and blocked with 5% nonfat dried milk in Tris-buffered saline containing 0.05% Tween-20 (TBST). Antibody dilutions and incubation conditions are given in Supplementary Methods. Proteins were visualized using Clarity Western ECL Blotting substrate (Bio-Rad), SuperSignal West Dura Extended Duration Substrate or SuperSignal West Femto Maximum Sensitivity Substrate (Thermo Scientific). The membranes were scanned using a UVP BioSpectrum AC Imaging System (UVP). Densitometry measurements were done using ImageJ software. Results were confirmed by at least two independent experiments.

### Affinity purification of 3XFLAG protein complexes

HeLa cells were reverse transfected with mammalian expression vectors for human 3XFLAG-SMN, 3XFLAG-SMN6B or 3XFLAG-hnRNP A1, using Lipofectamine 2000. Whole-cell lysates from transfected cells were prepared using NP-40 buffer (see Supplementary Methods). After centrifugation the supernatants were added to anti-FLAG beads (Sigma) equilibrated with NP-40 buffer (~1 mg of total proteins was used for each sample) and incubated for 2.5 hours with gentle mixing at 4 °C. Supernatants were then discarded and the beads were washed once with high salt buffer (20 mM Tris-HCl pH 7.5, 500 mM NaCl, 0.02% NP-40) followed by two washes with NP-40 buffer. Protein complexes were eluted with NP-40 buffer containing 0.25 mg/ml FLAG peptide (Sigma) for 1 hour at 8 °C and analyzed by SDS-PAGE.

### Immunofluorescence analysis

HeLa cells pre-plated on 12 mm glass coverslips were transfected with empty vector (pCI, Promega) or expression vectors for 3XFLAG-tagged proteins of interest. Cells were fixed with 4% paraformaldehyde (Polysciences, Inc). Cells were then washed with PBS, blocked with 5% normal goat serum (Cell Signaling), and overexpressed proteins were detected using Alexa-555-conjugated rabbit anti-DYKDDDDK (Cell Signaling). Nuclei were stained and coverslips mounted using Vectashield Mounting medium with DAPI (Vector Laboratories). Images were captured using a Leica SP5XMP microscope system at the Iowa State University Confocal and Multiphoton Facility. Captured confocal images were viewed/converted to TIFF files using LAS AF lite v2.6. For more detail see Supplementary Methods.

### Sequence analysis

The genomic sequence of *SMN1* was obtained from NCBI and repeat sequences were identified and classified using Repeatmasker (http://www.repeatmasker.org/). Illumina sequencing data of hnRNP C knockdown was originally described in Zarnack *et al.* and was obtained from ArrayExpress (Accession: E-MTAB-1147)^35^. Sequencing data of UPF1 knockdown was obtained from the Sequence Read Archive (Accession: SRP063462). Reads were mapped to the human genome version hg38 using TopHat^46^. For transcript identification, Gencode version 20 was used (http://www.gencodegenes.org). Mapped reads were visualized and junction reads were counted using Integrative Genomics Viewer^47^. To quantify relative inclusion of internal exons, reads which mapped across splice junctions were counted and percent inclusion was calculated by dividing the total number of inclusion-supporting reads by the total of all inclusion- and exclusion-supporting reads.

### Comparative Modeling

Representative models of the SMN6B protein and the dimeric YG domains of SMN∆7 and SMN6B were calculated using the RosettaCM protocol utilizing multiple structure templates for comparative modeling^48^. The amino acid sequence of SMN6B used for modeling is based on the DNA sequence of the *SMN2* splice variant that includes exon 6B. For comparative modeling, structure templates of the SMN domains were selected from the Protein Data Bank. Selected structure templates and detailed methods can be found in Supplementary Methods.

### Statistical analysis

Relative quantities with standard deviations were calculated by using ΔΔCT method by Excel (Microsoft Office 2011 edition). Data were expressed as mean ± standard error of the mean (SEM). Statistical analyses were performed using the unpaired Student’s *t*-test. Unless otherwise mentioned, *p* values were two-tailed and the level of statistical significance was set at *p* < 0.05.

## Additional Information

**How to cite this article**: Seo, J. *et al.* A novel human-specific splice isoform alters the critical C-terminus of Survival Motor Neuron protein. *Sci. Rep.* **6**, 30778; doi: 10.1038/srep30778 (2016).

## Supplementary Material

Supplementary Information

## References

[b1] Nilsen T. W. & Graveley B. R. Expansion of the eukaryotic proteome by alternative splicing. Nature 463, 457–463, doi: 10.1038/nature08909 (2010). 20110989 PMC3443858

[b2] Lander E. S. *et al.* Initial sequencing and analysis of the human genome. Nature 409, 860–921, doi: 10.1038/35057062 (2001). 11237011

[b3] Li W. H., Gu Z. L., Wang H. D. & Nekrutenko A. Evolutionary analyses of the human genome. Nature 409, 847–849, doi: 10.1038/35057039 (2001). 11237007

[b4] Sorek R., Ast G. & Graur D. Alu-containing exons are alternatively spliced. Genome Research 12, 1060–1067, doi: 10.1101/gr.229302 (2002). 12097342 PMC186627

[b5] Lin L. *et al.* Diverse Splicing Patterns of Exonized Alu Elements in Human Tissues. Plos Genetics 4, 13, doi: 10.1371/journal.pgen.1000225 (2008). PMC2562518 18841251

[b6] Keren H., Lev-Maor G. & Ast G. Alternative splicing and evolution: diversification, exon definition and function. Nature Reviews Genetics 11, 345–355, doi: 10.1038/nrg2776 (2010). 20376054

[b7] Schmitz J. & Brosius J. Exonization of transposed elements: A challenge and opportunity for evolution. Biochimie 93, 1928–1934, doi: 10.1016/j.biochi.2011.07.014 (2011). 21787833

[b8] Lewis B. P., Green R. E. & Brenner S. E. Evidence for the widespread coupling of alternative splicing and nonsense-mediated mRNA decay in humans. Proceedings of the National Academy of Sciences of the United States of America 100, 189–192, doi: 10.1073/pnas.0136770100 (2003). 12502788 PMC140922

[b9] Lykke-Anderson S. & Jensen T. H. Nonsense-mediated mRNA decay: an intricate machinery that shapes transcriptomes. Nature Reviews Molecular Cell Biology 16, 665–677, doi: 10.1038/nrm4063 (2015). 26397022

[b10] Karam R., Wengrod J., Gardner L. B. & Wilkinson M. F. Regulation of nonsense-mediated mRNA decay: Implications for physiology and disease. Biochimica Et Biophysica Acta-Gene Regulatory Mechanisms 1829, 624–633, doi: 10.1016/j.bbagrm.2013.03.002 (2013). PMC3660545 23500037

[b11] Lefebvre S. *et al.* Identification and characterization of a spinal muscular atrophy-determining gene. Cell 80, 155–165, doi: 10.1016/0092-8674(95)90460-3 (1995). 7813012

[b12] Lorson C. L., Hahnen E., Androphy E. J. & Wirth B. A single nucleotide in the SMN gene regulates splicing and is responsible for spinal muscular atrophy. Proceedings of the National Academy of Sciences of the United States of America 96, 6307–6311, doi: 10.1073/pnas.96.11.6307 (1999). 10339583 PMC26877

[b13] Tisdale S. & Pellizzoni L. Disease Mechanisms and Therapeutic Approaches in Spinal Muscular Atrophy. Journal of Neuroscience 35, 8691–8700, doi: 10.1523/jneurosci.0417-15.2015 (2015). 26063904 PMC4461682

[b14] Ahmad S., Bhatia K., Kannan & Gangwani L. Molecular mechanisms of neurodegeneration in Spinal Muscular Atrophy. Journal of Experimental Neuroscience 10, 39–49, doi: 10.4137/JEN.S33122 (2016). 27042141 PMC4807884

[b15] Zhang R. D. *et al.* Structure of a Key Intermediate of the SMN Complex Reveals Gemin2’s Crucial Function in snRNP Assembly. Cell 146, 384–395, doi: 10.1016/j.cell.2011.06.043 (2011). 21816274 PMC3160754

[b16] Zhao D. Y. *et al.* SMN and symmetric arginine dimethylation of RNA polymerase II C-terminal domain control termination. Nature 529, 48–53, doi: 10.1038/nature16469 (2016). 26700805

[b17] Sanchez G. *et al.* A novel function for the survival motoneuron protein as a translational regulator. Human Molecular Genetics 22, 668–684, doi: 10.1093/hmg/dds474 (2013). 23136128

[b18] Takaku M. *et al.* Purification of the Human SMN-GEMIN2 Complex and Assessment of Its Stimulation of RAD51-Mediated DNA Recombination Reactions. Biochemistry 50, 6797–6805, doi: 10.1021/bi200828g (2011). 21732698

[b19] Piazzon N. *et al.* Implication of the SMN complex in the biogenesis and steady state level of the Signal Recognition Particle. Nucleic Acids Research 41, 1255–1272, doi: 10.1093/nar/gks1224 (2013). 23221635 PMC3553995

[b20] Zou T. *et al.* SMN Deficiency Reduces Cellular Ability to Form Stress Granules, Sensitizing Cells to Stress. Cellular and Molecular Neurobiology 31, 541–550, doi: 10.1007/s10571-011-9647-8 (2011). 21234798 PMC11498399

[b21] Ahmad S., Wang Y., Shaik G. M., Burghes A. H. & Gangwani L. The zinc finger protein ZPR1 is a potential modifier of spinal muscular atrophy. Human Molecular Genetics 21, 2745–2758, doi: 10.1093/hmg/dds102 (2012). 22422766 PMC3363332

[b22] Branchu J. *et al.* Shift from Extracellular Signal-Regulated Kinase to AKT/cAMP Response Element-Binding Protein Pathway Increases Survival-Motor-Neuron Expression in Spinal-Muscular-Atrophy-Like Mice and Patient Cells. Journal of Neuroscience 33, 4280–4294, doi: 10.1523/jneurosci.2728-12.2013 (2013). 23467345 PMC6704952

[b23] Peter C. J. *et al.* The COPI vesicle complex binds and moves with survival motor neuron within axons. Human Molecular Genetics 20, 1701–1711, doi: 10.1093/hmg/ddr046 (2011). 21300694 PMC3071668

[b24] Fallini C. *et al.* The Survival of Motor Neuron (SMN) Protein Interacts with the mRNA-Binding Protein HuD and Regulates Localization of Poly(A) mRNA in Primary Motor Neuron Axons. Journal of Neuroscience 31, 3914–3925, doi: 10.1523/jneurosci.3631-10.2011 (2011). 21389246 PMC3070748

[b25] Howell M. D., Singh N. N. & Singh R. N. Advances in therapeutic development for spinal muscular atrophy. Future Medicinal Chemistry 6, 1081–1099, doi: 10.4155/fmc.14.63 (2014). 25068989 PMC4356243

[b26] Gupta K. *et al.* Oligomeric Properties of Survival Motor Neuron.Gemin2 Complexes. Journal of Biological Chemistry 290, 20185–20199, doi: 10.1074/jbc.M115.667279 (2015). 26092730 PMC4536428

[b27] Zhang H. L. L. *et al.* Active transport of the survival motor neuron protein and the role of exon-7 in cytoplasmic localization. Journal of Neuroscience 23, 6627–6637 (2003). 12878704 10.1523/JNEUROSCI.23-16-06627.2003 PMC6740639

[b28] Cho S. C. & Dreyfuss G. A degron created by SMN2 exon 7 skipping is a principal contributor to spinal muscular atrophy severity. Genes & Development 24, 438–442, doi: 10.1101/gad.1884910 (2010). 20194437 PMC2827839

[b29] Martin R., Gupta K., Ninan N. S., Perry K. & Van Duyne G. D. The Survival Motor Neuron Protein Forms Soluble Glycine Zipper Oligomers. Structure 20, 1929–1939, doi: 10.1016/j.str.2012.08.024 (2012). 23022347 PMC3519385

[b30] Ottesen E. W. *et al.* Severe impairment of male reproductive organ development in a low SMN expressing mouse model of spinal muscular atrophy. Scientific Reports 6, 20193, doi: 10.1038/srep20193 (2016). 26830971 PMC4735745

[b31] Singh N. N., Seo J., Rahn S. J. & Singh R. N. A Multi-Exon-Skipping Detection Assay Reveals Surprising Diversity of Splice Isoforms of Spinal Muscular Atrophy Genes. Plos One 7, 17, doi: 10.1371/journal.pone.0049595 (2012). PMC3501452 23185376

[b32] Osborne M. *et al.* Characterization of behavioral and neuromuscular junction phenotypes in a novel allelic series of SMA mouse models. Human Molecular Genetics 21, 4431–4447, doi: 10.1093/hmg/dds285 (2012). 22802075 PMC3459466

[b33] Gal-Mark N., Schwartz S. & Ast G. Alternative splicing of Alu exons - two arms are better than one. Nucleic Acids Research 36, 2012–2023, doi: doi: 10.1093/nar/gkn024 (2008). 18276646 PMC2330237

[b34] Park S. J. *et al.* Gain of a New Exon by a Lineage-Specific Alu Element-Integration Event in the BCS1L Gene during Primate Evolution. Molecules and Cells 38, 950–958, doi: 10.14348/molcells.2015.0121 (2015). 26537194 PMC4673409

[b35] Zarnack K. *et al.* Direct Competition between hnRNP C and U2AF65 Protects the Transcriptome from the Exonization of Alu Elements. Cell 152, 453–466, doi: 10.1016/j.cell.2012.12.023 (2013). 23374342 PMC3629564

[b36] Urano E., Miyauchi K., Ichikawa R., Futahashi Y. & Komano J. Regulation of cyclin T1 expression and function by an alternative splice variant that skips exon 7 and contains a premature termination codon. Gene 505, 1–8, doi: 10.1016/j.gene.2012.06.006 (2012). 22692005

[b37] Kurosaki T. & Maquat L. E. Nonsense-mediated mRNA decay in humans at a glance. Journal of Cell Science 129, 461–467, doi: 10.1242/jcs.181008 (2016). 26787741 PMC4760306

[b38] Kim S. *et al.* Transmembrane glycine zippers: Physiological and pathological roles in membrane proteins. Proceedings of the National Academy of Sciences of the United States of America 102, 14278–14283, doi: 10.1073/pnas.0501234102 (2005). 16179394 PMC1242278

[b39] Hua Y. M. & Zhou J. H. Survival motor neuron protein facilitates assembly of stress granules. Febs Letters 572, 69–74, doi: 10.1016/j.febslet.2004.07.010 (2004). 15304326

[b40] Kedersha N., Ivanov P. & Anderson P. Stress granules and cell signaling: more than just a passing phase? Trends in Biochemical Sciences 38, 494–506, doi: 10.1016/j.tibs.2013.07.004 (2013). 24029419 PMC3832949

[b41] Bish R. *et al.* Comprehensive Protein Interactome Analysis of a Key RNA Helicase: Detection of Novel Stress Granule Proteins. Biomolecules 5, 1441–1466, doi: 10.3390/biom5031441 (2015). 26184334 PMC4598758

[b42] Kelley D. R., Hendrickson D. G., Tenen D. & Rinn J. L. Transposable elements modulate human RNA abundance and splicing via specific RNA-protein interactions. Genome Biology 15, 537, doi: 10.1186/s13059-014-0537-5 (2014). 25572935 PMC4272801

[b43] Colak D., Ji S. J., Porse B. T. & Jaffrey S. R. Regulation of Axon Guidance by Compartmentalized Nonsense-Mediated mRNA Decay. Cell 153, 1252–1265, doi: 10.1016/j.cell.2013.04.056 (2013). 23746841 PMC3685487

[b44] Tajnik M. *et al.* Intergenic Alu exonisation facilitates the evolution of tissue-specific transcript ends. Nucleic Acids Research 43, 10492–10505, doi: 10.1093/nar/gkv956 (2015). 26400176 PMC4666398

[b45] Singh N. N. *et al.* TIA1 Prevents Skipping of a Critical Exon Associated with Spinal Muscular Atrophy. Molecular and Cellular Biology 31, 935–954, doi: 10.1128/mcb.00945-10 (2011). 21189287 PMC3067828

[b46] Trapnell C., Pachter L. & Salzberg S. L. TopHat: discovering splice junctions with RNA-Seq. Bioinformatics 25, 1105–1111, doi: 10.1093/bioinformatics/btp120 (2009). 19289445 PMC2672628

[b47] Robinson J. T. *et al.* Integrative genomics viewer. Nature Biotechnology 29, 24–26, doi: 10.1038/nbt.1754 (2011). PMC3346182 21221095

[b48] Song Y. F. *et al.* High-Resolution Comparative Modeling with RosettaCM. Structure 21, 1735–1742, doi: 10.1016/j.str.2013.08.005 (2013). 24035711 PMC3811137

