# Supplementary material for: A novel human-specific splice isoform alters the critical C-terminus of Survival Motor Neuron protein
Source: Sci Rep. 2016 Aug 2;6:30778. doi: 10.1038/srep30778 (PMC4969610; doi:10.1038/srep30778)
Supplement: Supplementary Information [file srep30778-s1.pdf]

A novel human-specific splice isoform alters the critical C-terminus of Survival Motor Neuron protein

Supplementary Figures, Table and Methods

Joonbae Seo, Natalia N. Singh, Eric W. Ottesen, Brian M. Lee and Ravindra N. Singh

Supplementary Figure Legends.

Supplementary Fig. 1. Alignment of SMN proteins from a variety of species. Corresponding human exons are demarcated on the top. Sequences were aligned by ClustalW algorithm using MacVector software. Letters in bold and highlighted in gray indicate consensus amino acids. Name of species are shown on the left; accession numbers are indicated at the end of each sequence. Gemin2-interacting domain and YG Box are demarcated.

Supplementary Fig. 2. Distribution of repetitive elements within *SMN* gene. Organization of *SMN* gene. Exons and introns are shown as rectangular boxes and lines, respectively. Arrows indicate orientations of sequences derived from transposable elements. Colors indicate different families of transposable elements, as indicated in the legend below.

Supplementary Fig. 3. Exon 6B is derived from an Alu conserved among many primates. **(a)** Alignment of *SMN* exon 6 and intron 6 regions spanning the AluY insertion of different primates. Hyphens designate the positions where gaps were introduced to maximize sequence identity. Colored boxes indicate exonic sequences. Red triangles indicate splice site positions. The orange arrow indicates position and direction of AluY insertion, left and right arms are indicated. Sequence alignment was performed using the ClustalW algorithm (EMBL-EBI). AluY sequences (reverse and complement) are obtained from Dfam (Accession number DF0000002). Abbreviations and GeneID numbers of all sequences used are as follows: Hu: Human, 6606; Chi: Chimpanzee, 461829; Gor: Gorilla, 101149971; Ora: Orangutan, 100171813; Mac: Macaque, 102130507. **(b)** Alignment of sequences immediately surrounding splice sites and estimated splice site strength calculated by the Maximum Entropy score[1]. Abbreviations are the same as in (a).

Supplementary Fig. 4. Effect of splicing factor knockdowns on exon 6B splicing. (a) Estimated exon inclusion for all internal exons of *SMN* based on RNA-Seq upon knockdown of various hnRNP proteins[2] (SRA accession number SRP010280). Values are expressed as percentage of all splice junction-crossing reads supporting exon inclusion. Error bars represent standard error of 2-6 sequencing libraries. **(b)** Estimated exon inclusion for all internal exons of

SMN based on RNA-Seq upon knockdown of PTB1[3] (SRA accession number SRP045065). Values are expressed as percentage of all splice junction-crossing reads supporting exon inclusion. Error bars represent standard error of 2 sequencing libraries. **(c)** Estimated exon inclusion for all internal exons of *SMN* based on RNA-Seq upon knockdown of TIA1/TIAR[4] (SRA accession number SRP021918). Values are expressed as percentage of all splice junction-crossing reads supporting exon inclusion. **(d)** Estimated exon inclusion for all internal exons of *SMN* based on RNA-Seq upon knockdown of HuR[5] (SRA accession number SRP007498) at 2 and 5 days post-knockdown. Values are expressed as percentage of all splice junction-crossing reads supporting exon inclusion.

Supplementary Fig. 5. SMN YG domain is similar to a glycine zipper. Representative YG domain models of SMNΔ7 and SMN6B are shown for comparison with structures containing a glycine zipper dimer interface. All structures are shown with stick representations for all heavy atoms and selected hydrogens. The carbon atoms of each chain within the dimer are colored separately, pale green for chain A and plum for chain B. Other atoms are colored by element with red for oxygen, blue for nitrogen, gold for sulfur and white for hydrogen. Hydrogen bonds are shown as yellow dashed lines. Selected residues are shown with side chain heavy atoms represented as van der Waals spheres. **(a)** In the SMNΔ7 model, the last residue in the $(YXXG)_3$ tetrad repeat of the YG domain has been replaced with Glu279, which distorts the dimeric coiled-coil interaction. **(b)** In the SMN6B model, the last residue of the YG domain is replaced with Thr279 creating both a distortion of the coiled-coil interface and a break in the helix structure. The potential destabilizing interactions between the bulky beta-carbon substituents of Thr279 and Cys283 contribute to breaking the helix. Both YG domain models have conserved glycine residues at the dimer interface that allow for close packing between helices and intra-helical side chain to backbone hydrogen bonds involving the conserved serine and threonine residues[6]. **(c)** A similar interaction motif, called a glycine zipper, was first seen in the NMR structure of the dimeric transmembrane domain of glycophorin A, PDB code: 1AFO[7]. The glycine zipper encompasses a conserved GXXXG sequence that is commonly found in membrane proteins and mediates interaction between transmembrane helices. **(d)** The glycine zipper also mediates dimerization of soluble proteins such as the Holliday junction endonuclease, resolvase, PDB code: 1HJR[8]. The alpha helix in the glycine zipper is slightly under wound and

shifts the coiled-coil interaction between the helices to right-handed supercoiling. The dimer interface may be stabilized by inter-molecular Cα-H···O backbone hydrogen bonds between each glycine and the *i*-3 residue in the other helix[9].

Supplementary Fig. 6. Exon 6B amino acid sequence is similar to sequences found in several predicted proteins. Diagrammatic representation of SMN6B protein derived from *SMN6B* transcript. Exons are shown as colored boxes, with their names given at the top. Number of amino acids encoded by each exon is given within each box. Location of the start and stop codons as well as 5′ and 3′ untranslated regions (UTRs) are indicated. The predicted amino acid sequence coded by exon 6B is given below, along with an alignment with similar sequences in NCBI's GenBank database. Similar sequences were identified by BLASTP using MacVector software. Stars signify sequence identity, while letters highlighted in red color indicate sequence differences. Names of the proteins are shown on the left; GenBank accession numbers are shown on the right with species names given in brackets.

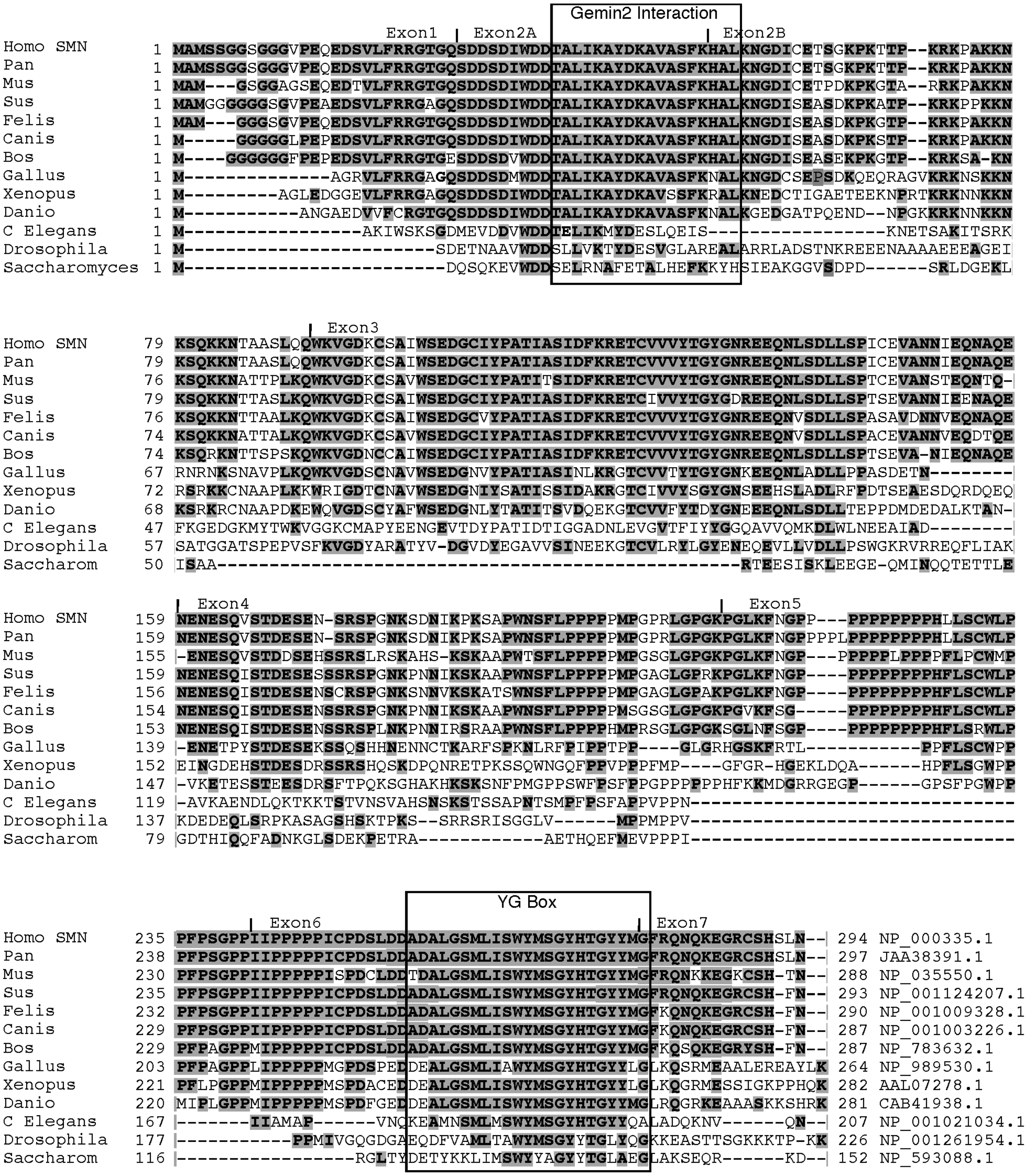

Supplementary Figure 1

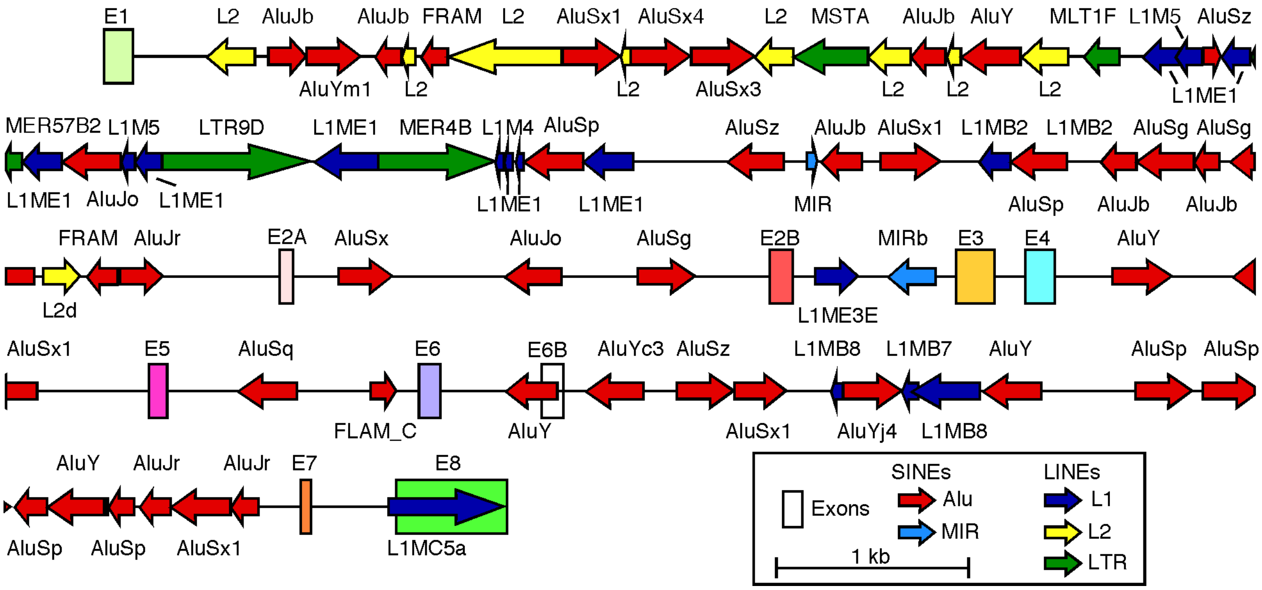

Supplementary Figure 2

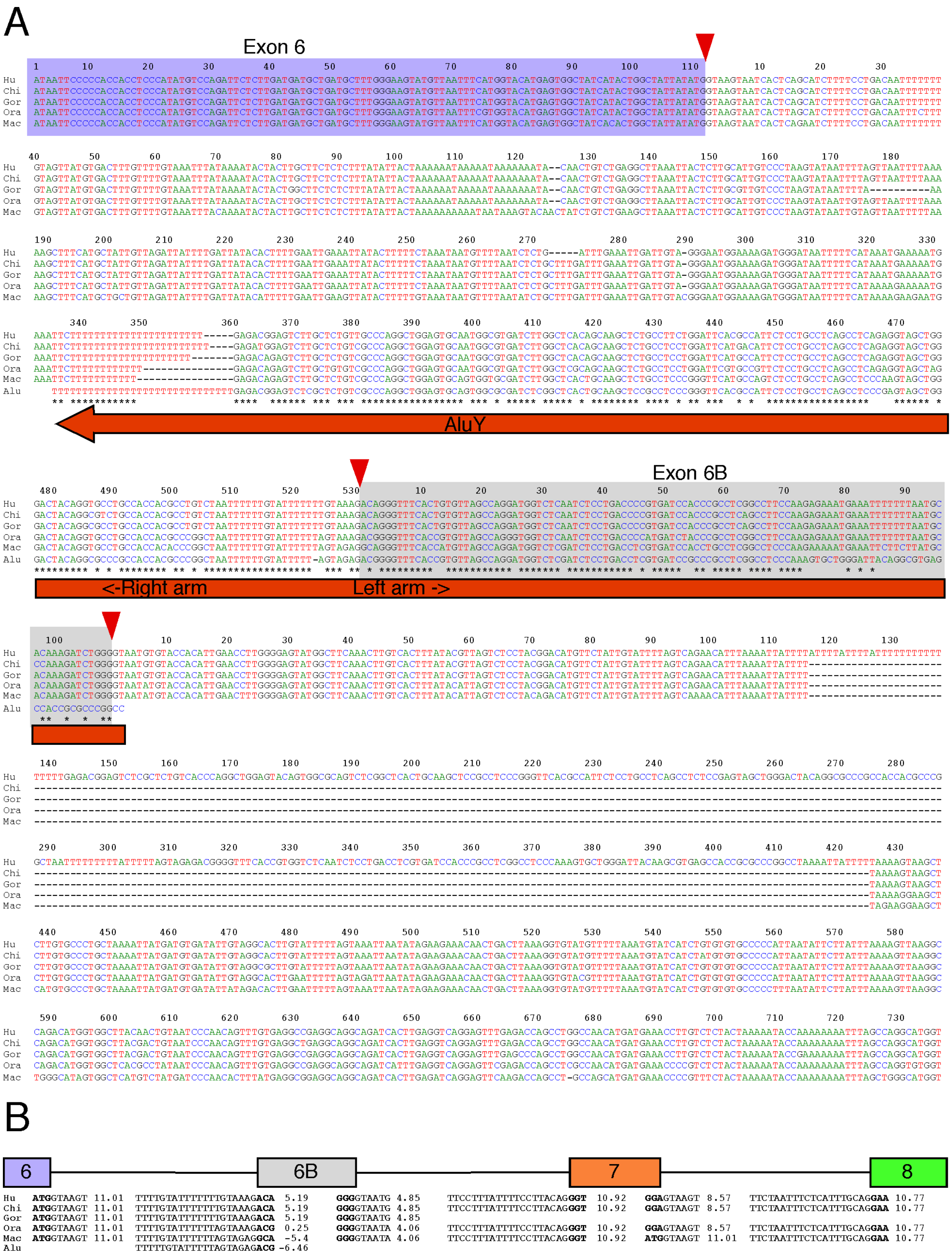

Supplementary Figure 3

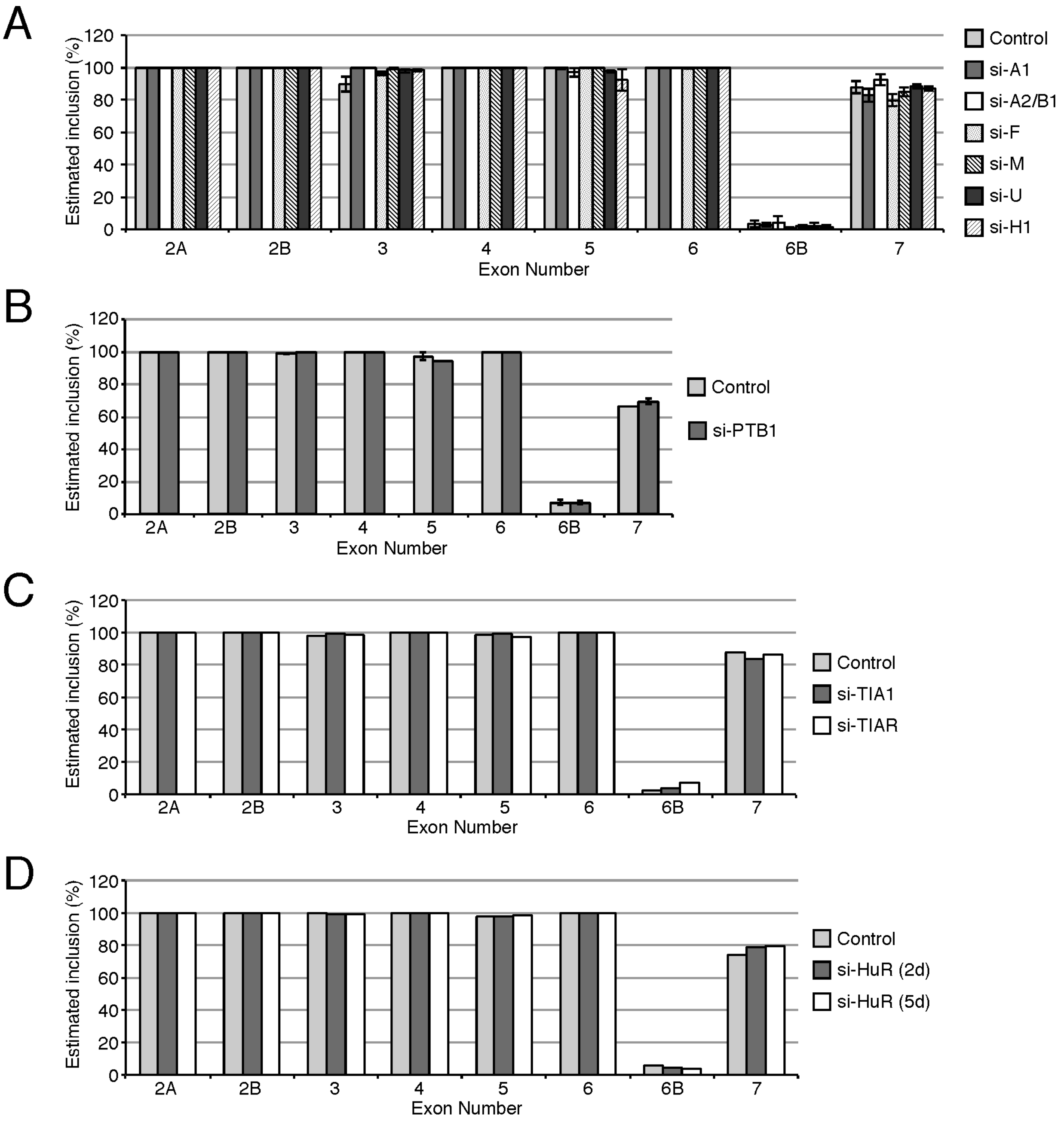

Supplementary Figure 4

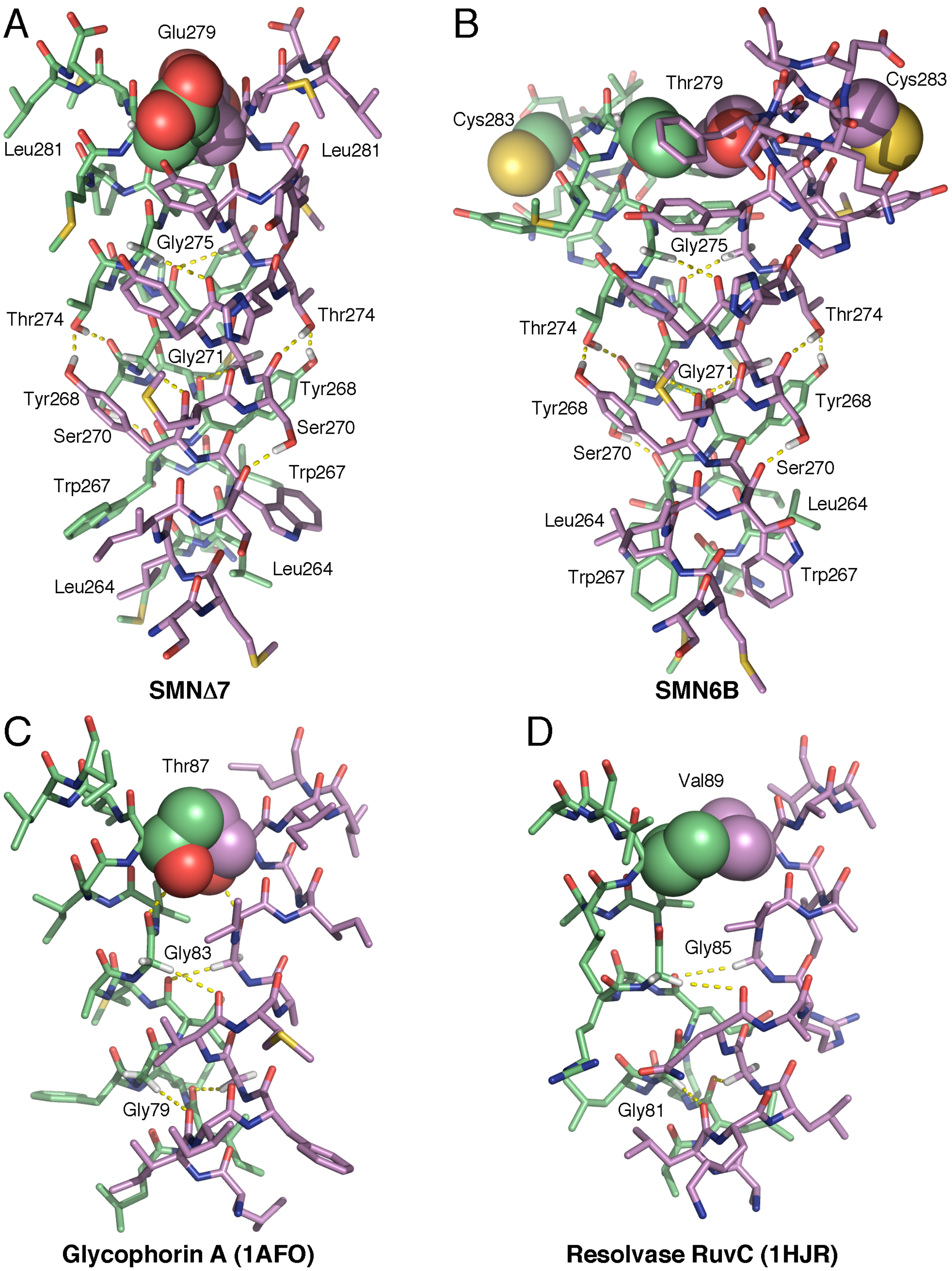

Supplementary Figure 5

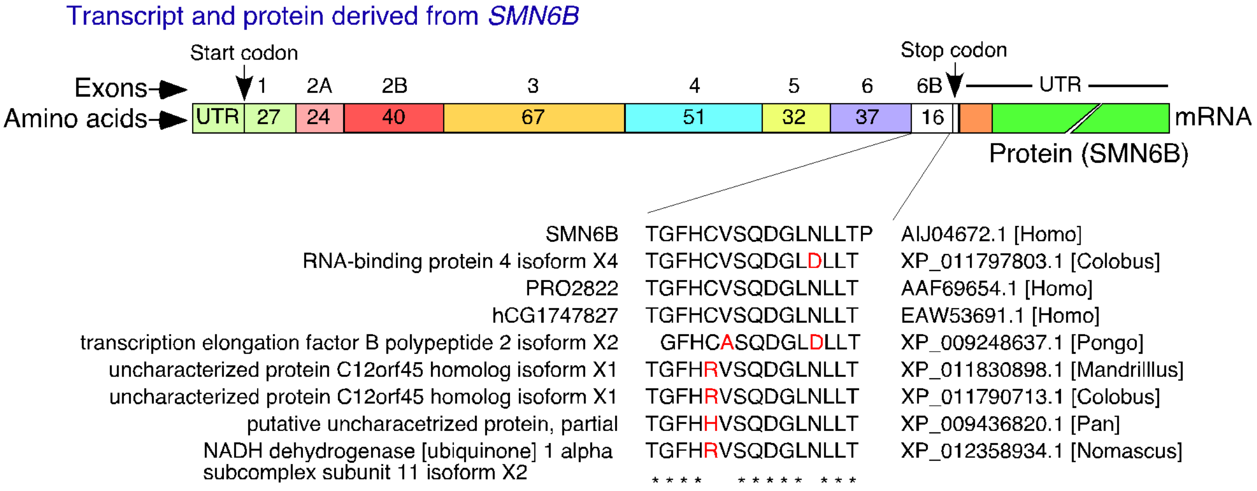

Supplementary Figure 6

Supplementary Table 1. Primers used in this study.

No.	Name	Type	Sequence (5′ to 3′)	Annealing site
1	P0	Forward	GAATACTGCAGCTTCCTTACAACAG	E2B
2	P2-2	Reverse	CTTCCTTTTTTCTTTCCCAACAC	E8
3	5′E6	Forward	CCTCCCATATGTCCAGATTCTC	E6
4	3′E6+7	Reverse	CTTTTTGATTTTGTCTAAAACC**CATATAAT**	Jxn E6/E7
5	3′E6+8	Reverse	TGCTCTATGCCAGCATTTC**CATAT**	Jxn E6/E8
6	3′E6+6B	Reverse	CTAACACAGTGAAACCCTGT**CATATAAT**	Jxn E6/E6B
7	5′E1	Forward	CGCGGGTTTGCTATGGCGAT	E1
8	3′E1+2A	Reverse	CAGAATCATCGCT**CTGGCCTGT**	Jxn E1/E2A
9	5′E6+6B	Forward	GGCTATCATACTGGCTATTATATG**ACAGG**	Jxn E6/E6B
10	3′E8-Dde	Reverse	CTACAACACCCTTCTCACAGCTC	E8
11	5′E4-97	Forward	GGCCAAGACTGGGACCAGG	E4
12	P2	Forward	*GCATGCAAG*CTTCCTTTTTTCTTTCCCAACAC	E8
13	CCNT16F	Forward	CAAGCAAGGACTTAGCACAGAC	*CCNT1* E6
14	CCNT19-2R	Reverse	CAAGATCTCCACACTGGTTAAGTTGC	*CCNT1* E9
15	N24	Forward	CCAGATTCTCTTGATGATGCTGATGCTTTGGG	E6
16	5′ACTB	Forward	AAAGACCTGTACGCCAACAC	Actin
17	3′ACTB	Reverse	GTCATACTCCTGCTTGCTGA	Actin
18	5′FLAG	Forward	GACTACAAAGACGACGATGAC	3XFLAG
19	5′NEO	Forward	CGAGAAAGTATCCATCATGGCTG	Neomycin phosphotrans-ferase
20	3′UTR	Reverse	CGAATTCTAGAGCTCGAGGCAGGTTTTTTTTT TTTTTTTTVN	PolyA
21	3′RT-Univ	Reverse	CGAATTCTAGAGCTCGAGGCAG	3′UTR adapter
22	3′E8-25	Reverse	TTAGTGCTGCTCTATGCCAGCATTT	E8

Abbreviations, E, Exon; Jxn, Junction; Extra sequences are shown in Italic; junction sites are in bold.

Supplementary Methods

Cell culture, transfection and treatment of cells

Primary patient fibroblasts were obtained from Coriell Cell Repositories. Primary fibroblasts were grown in MEM (catalog #10370) supplemented with 2 mM GlutaMAX-I (catalog #35050) and 15% fetal bovine serum (FBS). Human neuroblastoma SH-SY5Y (ATCC) cells were cultured in 1:1 mixture of Minimum Essential Medium (MEM, catalog #11095) and F12 (catalog #11765) medium supplemented with 10% FBS. HeLa cells were grown in Dulbecco's Modified Eagle's Medium (DMEM, catalog #11965) supplemented with 10% FBS.

Cycloheximide (CHX, Sigma Life Science) stock solution (100 mg/ml) was prepared in Dimethyl Sulfoxide (DMSO, Fisher Scientific). CHX treatment of cells of interests was performed as follows. To test whether 6B-containing *SMN* transcripts are susceptible to NMD, GM03813, HeLa and SH-SY5Y cells were pre-plated at 1.125×10^5, 2.9×10^5 and 2.2×10^6 cells per one well of a 6-well plate, respectively. Next day (~24 h later) CHX stock solution was added to culture media to a final concentration of 20 μg/ml. DMSO was used as a control treatment and was added using the same volume as CHX (0.2 μl/ml of medium). CHX treatment continued for 6 h, after what cells were washed with ice-cold PBS three times and TRIzol reagent (Life Technologies) was directly added to each well to initiate total RNA purification.

To test the stability of SMN protein isoforms HeLa cells were reverse-transfected with the mammalian expression vector of interest (3XFLAG-SMN, 3XFLAG-SMNΔ7 or 3XFLAG-SMN6B) using Lipofectamine 2000. Briefly, plasmid-Lipofectamine 2000 complexes were prepared following the manufacturer's suggestions. For each expression vector ~ 6.94×10^6 HeLa cells in suspension were combined with a given plasmid:Lipofectamine 2000 complex and plated into one 100 mm tissue culture dish; 12 μg of a given vector was used per dish. Eighteen hours later cells from each dish were re-plated into 12X60 mm tissue culture dishes. CHX treatment was initiated ~39 h after cell transfections, using CHX at final concentration of 20 μg/ml. Samples were collected immediately before treatment (0 h time point), as well as 4 h and 8 h after the beginning of treatment. Cells were washed with ice-cold PBS three times, scraped and

divided: part of cells was used for total RNA isolation using TRIzol and part for preparation of whole-cell lysates. For one sample cells from two 60 mm plates were pooled together.

For affinity purification of 3XFLAG-tagged proteins, HeLa cells were reverse-transfected with the expression vector of interest (human 3XFLAG-SMN, 3XFLAG-SMN6B or 3XFLAG-hnRNP A1) using Lipofectamine 2000. Briefly, plasmid-Lipofectamine 2000 complexes were prepared following the manufacturer's suggestions. Each complex was then combined with HeLa cell suspension containing ~1.38×10^7 cells in a total volume of 20 ml, and the cells were plated in two 100 mm dishes. 20 μg of each vector was used per one dish. ~6 h later the medium was changed with fresh one. ~24 h after transfection, HeLa cells were washed with ice-cold PBS three times and collected by scraping.

RT-PCR

Total RNA was isolated using TRIzol reagent (Life Technologies) following the manufacturer's recommendations, digested using RQ1 RNase-free DNase (Promega), then further purified using a Qiagen RNeasy Mini RNA purification kit or phenol:chloroform (OmniPur) extraction followed by ethanol precipitation. RNA concentration was measured using a BioMate 3 spectrophotometer (Thermo Scientific). RNA isolated from human tissue was purchased from Ambion (FirstChoice Human Total RNA Survey panel); samples from each tissue were pooled from three healthy individuals. cDNA was generated from either 0.5 μg (for human tissues) or 1.6-2.0 μg (for cell lines and mouse tissues) of total RNA in a 10 μl reaction using SuperScript III (Life Technologies) and oligo $(dT)_{12-18}$, oligo (dT) with adapter sequence, or a gene-specific primer (3′E8-Dde). cDNA was amplified using Taq DNA Polymerase (New England Biolabs) in the presence of either a 5′-end-P^{32}-labelled primer or a trace amount of [α-^{32}P] dATP (3,000 Ci/mmole; Perkin-Elmer). MESDA was performed as previously described[10,11]. In all cases PCR products were amplified and resolved on a native polyacrylamide gel. For DdeI and BglII digestion PCR products were purified by phenol:chloroform extraction and ethanol precipitation prior to overnight restriction digestion. Analysis and quantifications of splice products were performed using a FPL-5000 Image Reader and Multi Gauge software (Fuji Photo Film Inc). For quantitative real-time PCR (QPCR), reactions were carried out in 20 μl using 1X FastStart Universal SYBR Green Master Mix (Roche), 300 nM of each primer, and 1.5 μl of a 1:20

dilution of cDNA template in a Stratagene Mx3005P thermocycler. For negative controls, cDNA was synthesized in the absence of RTase or water was used as a template during PCR amplification. List of all primers used in PCR is given in Supplementary Table 1.

Whole-cell lysate preparation

Cells were washed with ice-cold PBS three times and collected by scraping. Cell pellets were either immediately used for making whole-cell lysates or were snap-frozen in liquid nitrogen and moved to -80°C for storage until further usage for preparation of whole-cell lysates. To prepare lysates, pellets were re-suspended in RIPA buffer (Boston Bioproducts) or NP-40 buffer (20 mM Tris-HCl pH 7.4, 150 mM NaCl, 1% NP-40) supplemented with HALT Protease and Phosphatase Inhibitor cocktail (Thermo Scientific). The cells were lysed on ice for 30 min. For protein stability assay, cell suspensions were briefly sonicated and then placed on ice for 15 min. The lysates were then centrifuged at 13,000 rpm for 20 min at 4 ˚C to remove cell debris. Protein concentration was measured using the Bradford assay (Bio-Rad protein assay).

Western blotting

The following primary and secondary antibodies were used: mouse monoclonal anti-SMN (BD Transduction Laboratories, 1:4000), horseradish-peroxidase-conjugated anti-FLAG (Sigma Life Science, 1:4000), mouse monoclonal anti-Gemin2 (Sigma Life Science, 1:400 o/n), mouse monoclonal anti-hnRNP A1 (Abcam, 1:4000), mouse polyclonal enriched anti-6B (ascites, 1:300 o/n or IgG purified), rabbit polyclonal anti-Actin (Sigma, 1:2000), horseradish-peroxidase-conjugated secondary antibodies against mouse (Jackson immunoResearch, 1:5000) and rabbit (GE Healthcare, 1:2000). Often membranes were stripped using Restore Western Stripping Buffer (Thermo Scientific) and re-probed.

Generation of polyclonal antibodies (anti-6B-001) and purification of Immunoglobulin G

A cysteine was added toward the N-terminus of the synthetic peptide for conjugation with keyhole limpet haemocyanin (KLH). The 6B polypeptide was then conjugated to KLH using Imject^{TM} Maleimide-Activated mcKLH Spin Kit (Thermo Scientific). Mice were injected with 25 µg of KLH-linked 6B polypeptide by intraperitoneal (IP) route on days 1, 14 and 28 days followed by a bleed on day 42. At this time the sera was tested for immunogenicity and found to

have a low response. A fourth injection was then administered, followed by a second bleed 14 days later. The sera was then retested and found to have a high enough response (1:500). Mice were then primed using Pristane (Sigma), and after 5 days administered an IP injection of SP2/O cells. After 10 days, mice were euthanized and polyclonal fluids were collected.

Immunoglobulin (IgG) was purified from the polyclonal fluids with Protein A column (Pierce). Briefly, 2 ml of the polyclonal fluids was diluted 1:1 with Protein A IgG Binding buffer (Pierce) and the applied to a Protein A column. After adding 1 ml protein A plus Agarose, the samples were incubated with Agarose for 2 h with rotating at room temperature. Following the incubation the Agarose were washed three times by rotating for 10 min at room temperature. After washing, IgG was eluted with 1 ml IgG elution buffer (0.1 M glycine pH 3.0), then was immediately adjusted to physiologic pH by adding 100 µl neutralization buffer (1 M Tris, pH 8.5) per 1 ml of eluate. The eluded antibody was pooled and dialyzed in PBS. The purity of purified IgG was confirmed by 11% SDS-PAGE with Coomassie Blue staining. Antibodies were stored at -80°C.

Immunofluorescence analysis

HeLa cells were plated at a density of ~$1.76x10^5$ cells per one well of a 24-well plate on 12 mm glass coverslips (Bellco Glass, Inc). Next day the cells were transfected with 0.15 µg of an empty vector (pCI, Promega) or the expression vector for human 3XFLAG-SMN full length, Δ7 or 6B using X-tremeGENE HP DNA Transfection Reagent (Roche) following the manufacturer's instructions. ~24 h later the transfected cells were washed with PBS and fixed for 15 min with 4% paraformaldehyde solution freshly prepared by diluting Ultra Pure EM Grade 16% formaldehyde (Polysciences, Inc) in 1XPBS (pH 8.0). Fixed cells were then washed with 1XPBS 3 times, 5 min each, and blocked in the solution containing 1XPBS, 5% normal goat serum (Cell Signaling), and 0.3% Triton X-100 (Sigma Life Science) for 1 h at room temperature. Blocking solution was then changed with solution containing Alexa-555-conjugated rabbit primary antibody against DYKDDDDK Tag (Cell Signaling). The antibody was diluted 1:50 in the buffer containing 1XPBS, 0.1% Bovine Serum Albumin (BSA, Cell Signaling) and 0.3% Triton X-100. Incubation of the cells with the antibodies was carried overnight at 4°C. Next morning cells were extensively washed with 1XPBS followed by mounting on glass slides using Vectashield

Mounting medium with DAPI (Vector Laboratories). Subsequently coverslips were permanently sealed around the perimeter with nail polish.

Comparative Modeling

A representative model of SMN6B protein was calculated using the RosettaCM protocol[12]. The amino acid sequence of SMN6B used for modeling is based on the DNA sequence of the *SMN2* splice variant that includes the exon 6B. Multiple structure templates for each domain in SMN were selected from the Protein Data Bank (PDB). For each template sequence, an alignment with the SMN6B protein was prepared with Jalview and converted to Grishin format[13]. For the Gemin2 Binding domain of SMN, the structure templates included the crystal structure of the Gemin2-SMN_{Ge2BD}-Sm pentamer (D1/D2/F/E/G) complex, PDB code: 3S6N; and the solution NMR structure of the core SMN-Gemin2 complex, PDB code: 2LEH[14,15]. The templates for the Tudor domain of SMN included the solution NMR structure, PDB code: 1G5V; the crystal structure, PDB code: 1MHN; and a crystal structure bound to a small organic molecule, PDB code: 4QQ6[16-18]. For the YG domain, one protomer from each of the MBP fusion YG domain crystal structures was used as the structure template derived from either the human SMN YG domain, PDB code: 4GLI; or the yeast SMN YG domain, PDB code: 4RG5[6,19]. For modeling of the remaining regions of SMN6B, 3 and 9 residue fragment libraries were derived by a sequence-based search of the Protein Data Bank using the Robetta server[20]. The Grishin format sequence alignments were used for threading the SMN6B sequence into each structure template to generate partial models. The threaded models and fragment libraries were used to generate 100 hybrid models derived from multiple templates through low-resolution Monte Carlo based sampling and quasi-Newton minimization to optimize loop closure and backbone geometry. Selected models with intact YG Box helices were further refined using a full-atom realistic energy landscape and the lowest energy model was selected as the representative model of the SMN6B protein.

Models of the YG domain of SMNΔ7 and SMN6B isoforms were calculated with C2 symmetry using both the human and yeast YG domain crystal structures as templates[6,19]. The C2 symmetry definition was generated with the make_symmdef_file application using the yeast SMN YG domain structure (PDB code: 4RG5) and the noncrystallographic (point) symmetry option[21]. The

sequences for comparative modeling were restricted to the C-terminal residues that correlate to the predicted YG domain including residues 252-282 for the SMNΔ7 isoform and residues 252-294 for the SMN6B isoform. After threading these sequences into the YG domain structure templates, the hybridize application was used to calculate 10 models for each construct. The hybrid models were screened for dimer formation and an intact YG Box helix. Selected hybrid models were further refined to generate representative models of the coiled-coil dimers for both the SMNΔ7 and SMN6B YG domains.

Supplementary References

1 Yeo, G. & Burge, C. B. Maximum entropy modeling of short sequence motifs with applications to RNA splicing signals. *J Comput Biol* **11**, 377-394, doi:10.1089/1066527041410418 (2004).

2 Huelga, S. C. *et al.* Integrative Genome-wide Analysis Reveals Cooperative Regulation of Alternative Splicing by hnRNP Proteins. *Cell Reports* **1**, 167-178, doi:10.1016/j.celrep.2012.02.001 (2012).

3 Ge, Z., Quek, B. L., Beemon, K. L. & Hogg, J. R. Polypyrimidine tract binding protein 1 protects mRNAs from recognition by the nonsense-mediated mRNA decay pathway. *eLife* **5**, doi:10.7554/eLife.11155 (2016).

4 Nunez, M., Sanchez-Jimenez, C., Alcalde, J. & Izquierdo, J. M. Long-Term Reduction of T-Cell Intracellular Antigens Reveals a Transcriptome Associated with Extracellular Matrix and Cell Adhesion Components. *PLoS One* **9**, e113141, doi:10.1371/journal.pone.0113141 (2014).

5 Lebedeva, S. *et al.* Transcriptome-wide Analysis of Regulatory Interactions of the RNA-Binding Protein HuR. *Molecular Cell* **43**, 340-352, doi:10.1016/j.molcel.2011.06.008 (2011).

6 Martin, R., Gupta, K., Ninan, N. S., Perry, K. & Van Duyne, G. D. The survival motor neuron protein forms soluble glycine zipper oligomers. *Structure* **20**, 1929-1939, doi:10.1016/j.str.2012.08.024 (2012).

7 MacKenzie, K. R., Prestegard, J. H. & Engelman, D. M. A transmembrane helix dimer: structure and implications. *Science* **276**, 131-133, doi:10.1126/science.276.5309.131 (1997).

8 Ariyoshi, M. *et al.* Atomic structure of the RuvC resolvase: a holliday junction-specific endonuclease from E. coli. *Cell* **78**, 1063-1072, doi:10.1016/0092-8674(94)90280-1 (1994).

9 Kleiger, G., Grothe, R., Mallick, P. & Eisenberg, D. GXXXG and AXXXA: common alpha-helical interaction motifs in proteins, particularly in extremophiles. *Biochemistry* **41**, 5990-5997, doi:10.1021/bi0200763 (2002).

10 Seo, J., Ottesen, E. W. & Singh, R. N. Antisense Methods to Modulate Pre-mRNA Splicing. *Methods Mol Biol* **1126**, 271-283, doi:10.1007/978-1-62703-980-2_20 (2014).

11 Singh, N. N., Seo, J., Rahn, S. J. & Singh, R. N. A multi-exon-skipping detection assay reveals surprising diversity of splice isoforms of spinal muscular atrophy genes. *PLoS One* **7**, e49595, doi:10.1371/journal.pone.0049595 (2012).

12 Song, Y. *et al.* High-resolution comparative modeling with RosettaCM. *Structure* **21**, 1735-1742, doi:10.1016/j.str.2013.08.005 (2013).

13 Waterhouse, A. M., Procter, J. B., Martin, D. M., Clamp, M. & Barton, G. J. Jalview Version 2-a multiple sequence alignment editor and analysis workbench. *Bioinformatics* **25**, 1189-1191, doi:10.1093/bioinformatics/btp033 (2009).

14 Zhang, R. *et al.* Structure of a key intermediate of the SMN complex reveals Gemin2's crucial function in snRNP assembly. *Cell* **146**, 384-395, doi:10.1016/j.cell.2011.06.043 (2011).

15 Sarachan, K. L. *et al.* Solution structure of the core SMN-Gemin2 complex. *Biochem J* **445**, 361-370, doi:10.1042/BJ20120241 (2012).

16 Selenko, P. *et al.* SMN tudor domain structure and its interaction with the Sm proteins. *Nat Struct Biol* **8**, 27-31, doi:10.1038/83014 (2001).

17 Sprangers, R., Groves, M. R., Sinning, I. & Sattler, M. High-resolution X-ray and NMR structures of the SMN Tudor domain: conformational variation in the binding site for symmetrically dimethylated arginine residues. *J Mol Biol* **327**, 507-520, doi:10.1016/S0022-2836(03)00148-7 (2003).

18 Liu, Y. *et al.* Crystal Structure of Tudor Domain of Smn1 in Complex With a Small Organic Molecule. *Structural Genomics Consortium* (2014).

19 Gupta, K. *et al.* Oligomeric Properties of Survival Motor Neuron-Gemin2 Complexes. *J Biol Chem* **290**, 20185-20199, doi:10.1074/jbc.M115.667279 (2015).

20 Kim, D. E., Chivian, D. & Baker, D. Protein structure prediction and analysis using the Robetta server. *Nucleic Acids Res* **32**, W526-531, doi:10.1093/nar/gkh468 (2004).

21 DiMaio, F., Leaver-Fay, A., Bradley, P., Baker, D. & Andre, I. Modeling symmetric macromolecular structures in Rosetta3. *PLoS One* **6**, e20450, doi:10.1371/journal.pone.0020450 (2011).
